# Combined use of protein biomarkers and network analysis unveils deregulated regulatory circuits in Duchenne muscular dystrophy

**DOI:** 10.1371/journal.pone.0194225

**Published:** 2018-03-12

**Authors:** Silvia Parolo, Luca Marchetti, Mario Lauria, Karla Misselbeck, Marie-Pier Scott-Boyer, Laura Caberlotto, Corrado Priami

**Affiliations:** 1 The Microsoft Research—University of Trento Centre for Computational and Systems Biology (COSBI), Rovereto (TN), Italy; 2 Department of Mathematics, University of Trento, Povo (TN), Italy; 3 Department of Computer Science, University of Pisa, Pisa (PI), Italy; Universidade de Mogi das Cruzes, BRAZIL

## Abstract

Although the genetic basis of Duchenne muscular dystrophy has been known for almost thirty years, the cellular and molecular mechanisms characterizing the disease are not completely understood and an efficacious treatment remains to be developed. In this study we analyzed proteomics data obtained with the SomaLogic technology from blood serum of a cohort of patients and matched healthy subjects. We developed a workflow based on biomarker identification and network-based pathway analysis that allowed us to describe different deregulated pathways. In addition to muscle-related functions, we identified other biological processes such as apoptosis, signaling in the immune system and neurotrophin signaling as significantly modulated in patients compared with controls. Moreover, our network-based analysis identified the involvement of FoxO transcription factors as putative regulators of different pathways. On the whole, this study provided a global view of the molecular processes involved in Duchenne muscular dystrophy that are decipherable from serum proteome.

## Introduction

Proteomics, the large scale analysis of proteins, is feasible thanks to the availability of different technologies, such as mass spectrometry, gel-based techniques, antibody-based arrays and recently developed aptamer-based technologies [1–3]. Despite these technological improvements, the extraction of knowledge from the data produced is still challenging and the development of data analysis workflows capable of providing additional insight compared to traditional methods would be highly advantageous. In particular, a first desirable outcome is the identification of accurate diagnostic and prognostic markers suitable for subject stratification, which would shorten the path to the application of precision medicine concepts to the clinical practice [4–6]. Another highly desirable outcome is a better understanding of the disease on the basis of the newly available proteomic profiles. For this second aim a systems biology approach based on network analysis would enable integration of highly descriptive disease biomarkers with existing knowledge and potentially provide additional insight on the affected biological processes [7–9]. In this work we introduce an approach to achieve both these aims using recently published proteomics data obtained from a Duchenne Muscular Dystrophy (DMD) cohort. DMD is a rare disease caused by mutations in the gene that encodes dystrophin. The main clinical feature observed in DMD patients is the presence of muscular damage coupled with chronic inflammation that leads to a progressive muscular degeneration and fibrosis. The average age of diagnosis is usually at four-five years, when the symptoms appear and the disability starts to arise [10]. The availability of disease biomarkers that can be assessed using simple, non-invasive techniques would be useful to better understand the biological pathways altered in the disease and could be used for an early diagnosis. To this second end, serum creatine kinase (CK) level is usually evaluated. However, blood CK concentration shows high variability because it is influenced by age of the child, physical activity extent and pharmacological treatments [11]. In our study we employed an optimized version of the rank-based classification algorithm introduced in [12,13] to identify two biomarker panels based on SomaLogic profiles of DMD patients and controls and characterize their classification performance. We then used the panels as the starting point of an analysis aimed at identifying the biological pathways altered in the disease. To circumvent the limitation represented by the relatively short list of proteins measurable in blood with the SomaLogic array, we resorted to a network-based systems biology approach to identify a list of biological processes that are affected in DMD. Therefore a first contribution of our study is the identification of accurate diagnostic and prognostic markers suitable for subject stratification. Another contribution is a better understanding of the disease in terms of pathways identified as affected on the basis of the altered serum proteomic profiles.

## Results

### Workflow overview

**Fig 1:**
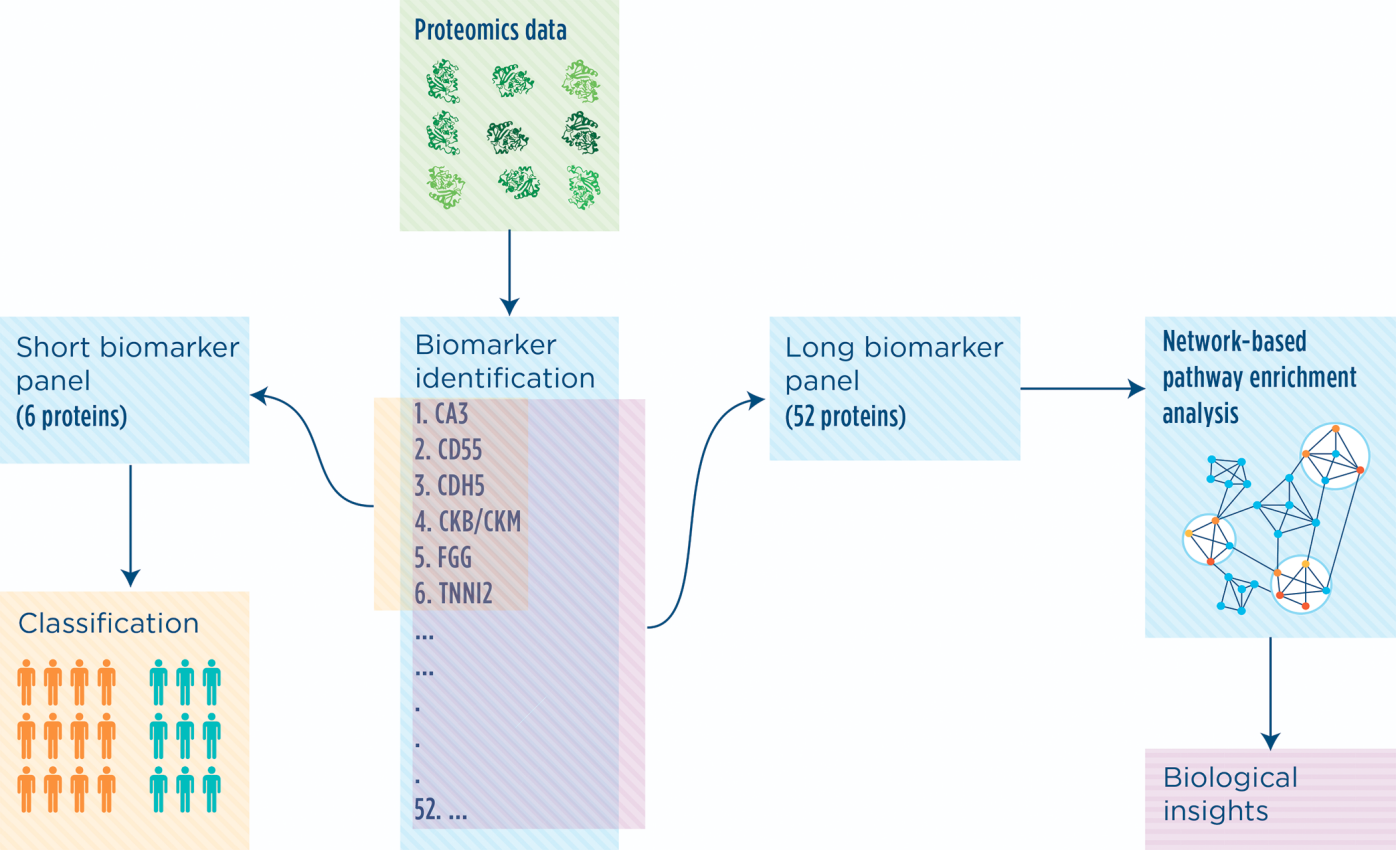
Study workflow. Proteomics data produced with SOMAscan technology were analyzed using a rank-based classification algorithm. We obtained two set of proteins (biomarker panels) useful for subject classification and disease characterization. The longer biomarker panel was used as input for network-based analysis.

We built a workflow based on a biomarker discovery algorithm and a tool for network analysis that we recently developed [12–14] (Fig 1). The biomarker discovery algorithm identifies subject-specific lists of proteins, herein called signatures, which are then compared in order to classify each subject as belonging to one of the two classes (affected or controls). The aggregation of the individual signatures produces a list of proteins useful for subject classification and we refer to it as a ‘biomarker panel’. Since the method can be tuned to obtain panels of different lengths, we applied it twice with different settings to obtain two biomarker panels, as shown in Fig 1. The shorter one was obtained specifically for classification purposes, while the longer one was derived to identify the biological processes affected by the disease. Specifically, we used the proteins in the long biomarker panel as input to the network-based tool NASFinder that identified functional modules from the sub-networks connecting biomarker proteins to known transcription factors.

### Identification of a short biomarker panel that discriminate DMD patients from controls

**Fig 2:**
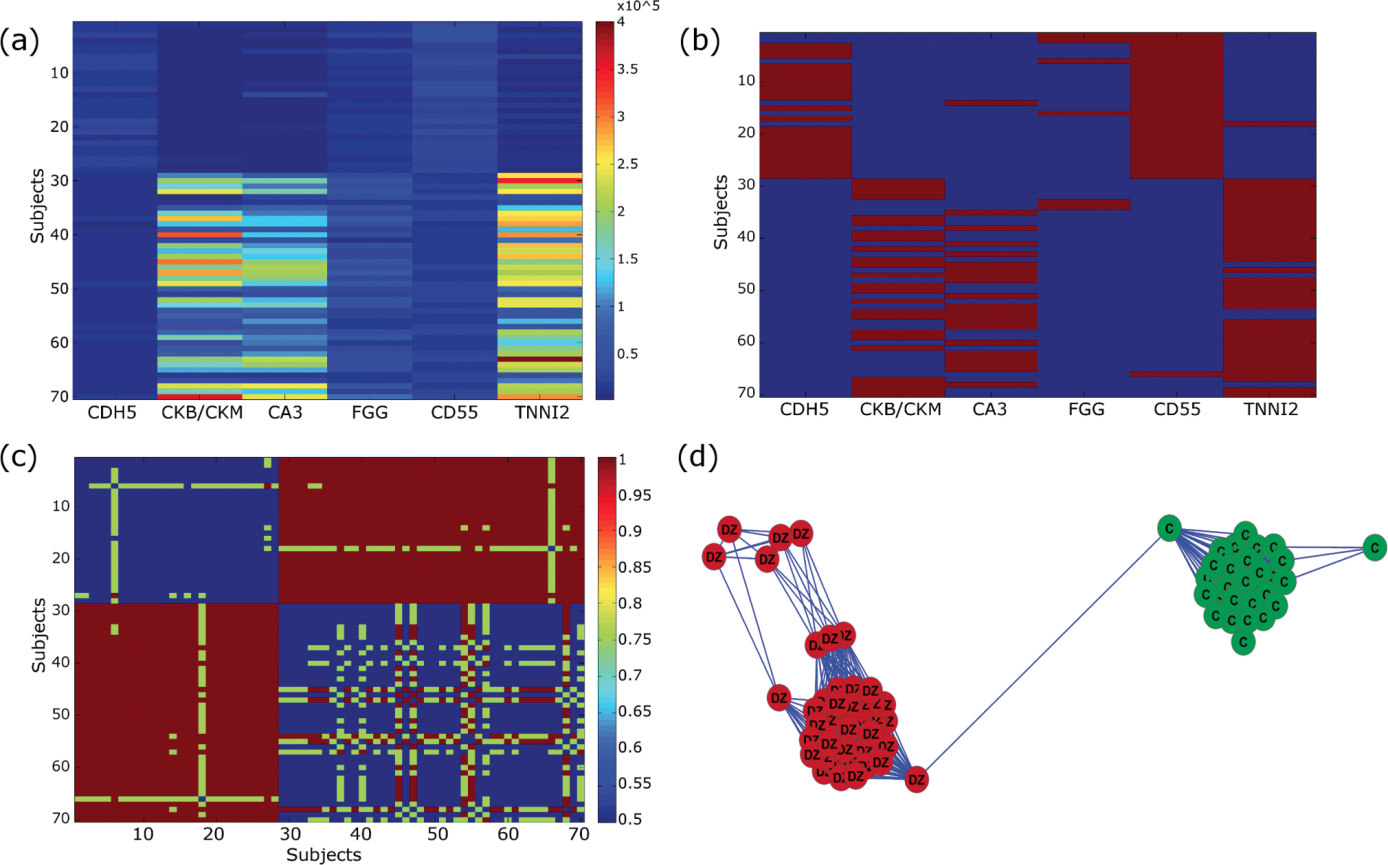
Signature-based classification of affected vs. control subjects for each individual. (A) Heatmap of the six proteins included in the biomarker panel (*columns*) across the 70 subjects (*rows*). Control subjects: top 28 rows; affected subjects: bottom 52 rows. (B) Signatures composed of at least two proteins (*red boxes*) out of six are needed to accurately classify each subject as being a member of either the control or the affected group. (C) The heatmap of the distance matrix shows that signatures of length two are actually sufficient to correctly divide subjects into two groups. (D) A map of the subjects based on the distance matrix confirms that the two emerging groups are of homogeneous composition and points to a possible subgroup of affected individuals (*green*: control subjects, *red*: affected subjects; colors were added after the map was drawn).

To identify biomarker panels associated with DMD, we analyzed concentration levels of 1128 blood proteins obtained using the SOMAscan technology on a cohort of 42 cases and 28 controls from The Parent Project Muscular Dystrophy-Cincinnati Children’s Hospital Medical Centre study (see details in Material and Methods). Overall, we determined that individual signatures formed of just two proteins out of a panel of six were satisfactory to discriminate DMD patients from controls with perfect accuracy (averaged over all the cross-validation rounds) and permutation test p-value < 0.001. These six protein epitopes correspond to the following genes: *CA3*, *CD55*, *CDH5*, *CKB/CKM*, *FGG* and *TNNI2* (Table 1). Principal component analysis performed on the dataset containing only data about these six proteins confirmed the separation between DMD patients and controls according to the main axis of variation (S1 Fig). The contribution of each protein in achieving a control/affected classification is illustrated in Fig 2. The heatmap of the measured protein levels shows that no single protein level highly correlates with a partition of the subjects in two groups (Fig 2A). Signatures of length two, as those selected by our method for inclusion in the short panel (Fig 2B, red boxes), are sufficient to correctly identify the control/affected status of each individual. As an intermediate step, our algorithm computes a complete distance matrix based on the similarity between each pair of subject signatures (shown in the form of a heatmap in Fig 2C). As an aid in interpreting the distance matrix, a map of the subjects is drawn by using the shortest N = 20% distances, with colors added afterwards according to control/affected status. In this case, the map clearly shows the emergence of two well-defined groups, with subject perfectly segregated by disease status (Fig 2D).

**Table 1:** Short biomarker panel.

SomaLogic ID	UniProt ID	Protein name*	Gene Symbol	p-value
3799–11_2	P07451	Carbonic anhydrase 3	CA3	1.31E-10
5069–9_3	P08174	Complement decay-accelerating factor	CD55	3.89E-09
2819–23_2	P33151	Cadherin-5	CDH5	2.30E-09
3714–49_2	P12277 / P06732	Creatine kinase B-type / Creatine kinase M-type	CKB CKM	1.32E-10
4989–7_1	P02679	Fibrinogen gamma chain	FGG	2.77E-09
5440–26_3	P48788	Troponin I, fast skeletal muscle	TNNI2	1.28E-10

* From Uniprot database

The six proteins are related to different fundamental aspects of the disease. In particular, serum creatine kinase is a marker of muscular damage [15] and it has been known for a long time for being increased in DMD patients, even if it is not DMD-specific [16]. Similarly, carbonic anhydrase 3 is another indicator of muscular damage and it is highly expressed in skeletal muscle. Also troponin is a muscle–specific protein involved in regulating muscle contraction and it was found in blood after prolonged exercise [15]. In addition to these proteins with a muscle origin, the biomarker panel included *CD55*, which encodes a regulator of complement cascade and, together with fibrinogen and cadherin, is likely involved in fibrosis, a DMD hallmark [17,18].

### Identification of a longer biomarker panel to investigate the disease biology

Despite the usefulness of the six proteins in the biomarker panel for classification purposes, they showed a limited applicability in understanding the pathways altered in the disease since only key aspects of the disease could be identified. We speculated that a longer list would be more likely to highlight the set of impaired pathways in a statistically significant way. For this reason, we trained the classification algorithm using different settings to obtain the longest protein list allowing classification accuracy close to 100% and we identified 52 proteins that are able to discriminate between disease and healthy controls with accuracy of prediction of 98.75% and permutation test p-value < 0.001 (Table 2).

**Table 2:** Long biomarker panel.

SomaLogic ID	UniProt ID	Gene Symbol	p-value
5451–1_3	Q13740	ALCAM	5.89E-07
4194–26_3	Q92688	ANP32B	3.27E-09
3799–11_2	P07451	CA3	1.68E-10
3326–58_2	Q9BY67	CADM1	7.29E-07
3350–53_2	Q9UQM7	CAMK2A	9.83E-10
3351–1_1	Q13554	CAMK2B	5.35E-08
3419–49_2	Q13557	CAMK2D	5.51E-09
3290–50_2	Q6YHK3	CD109	7.00E-07
5103–30_3	Q8TD46	CD200R1	3.00E-07
5069–9_3	P08174	CD55	6.39E-09
5337–64_3	P42081	CD86	2.78E-07
2819–23_2	P33151	CDH5	2.81E-09
3714–49_2	P12277 P06732	CKB CKM	6.67E-10
2670–67_4	P06732	CKM	1.50E-09
2827–23_2	P78423	CX3CL1	1.32E-07
4545–53_3	Q96DA6	DNAJC19	1.45E-07
2677–1_1	P00533	EGFR	1.55E-06
4908–6_1	P17813	ENG	4.88E-08
4696–2_2	P05413	FABP3	1.52E-09
5029–3_1	Q12884	FAP	1.46E-06
3052–8_2	P48023	FASLG	1.75E-06
4907–56_1	P02671 P02675 P02679	FGA FGB FGG	5.03E-09
2796–62_2	P02671 P02675 P02679	FGA FGB FGG	9.31E-09
4989–7_1	P02679	FGG	4.31E-09
2765–4_3	O95390	GDF11	3.84E-07
4272–46_2	P06744	GPI	8.07E-10
3709–4_2	P24298	GPT	3.57E-09
4775–34_3	P06396	GSN	6.53E-08
4553–65_3	Q7Z4V5	HDGFRP2	5.49E-07
4232–19_2	P08069	IGF1R	7.63E-07
3073–51_2	O95998	IL18BP	1.58E-06
5092–51_3	P78504	JAG1	1.51E-07
2475–1_3	P10721	KIT	2.48E-07
3890–8_2	P07195	LDHB	3.05E-08
5005–4_1	P53778	MAPK12	2.12E-10
3042–7_2	P02144	MB	9.01E-10
3853–56_1	P40925	MDH1	9.10E-07
5107–7_2	P46531	NOTCH1	7.60E-07
4179–57_3	None	None	1.65E-08
3390–72_2	P42336 P27986	PIK3CA PIK3R1	2.63E-08
2692–74_2	P14555	PLA2G2A	1.39E-07
2212–69_1	P00750	PLAT	1.60E-07
2961–1_2	P04070	PROC	5.45E-07
2696–87_2	O60542	PSPN	1.07E-06
5115–31_3	Q969Z4	RELT	4.87E-08
3220–40_2	P07949	RET	7.67E-10
3864–5_2	P62081	RPS7	7.06E-09
5122–92_2	Q9H2E6	SEMA6A	9.31E-07
2665–26_2	Q02223	TNFRSF17	4.19E-07
4472–5_2	P07951	TPM2	1.26E-06
5440–26_3	P48788	TNNI2	3.88E-10
5441–67_3	P19429	TNNI3	2.31E-09

**Fig 3:**
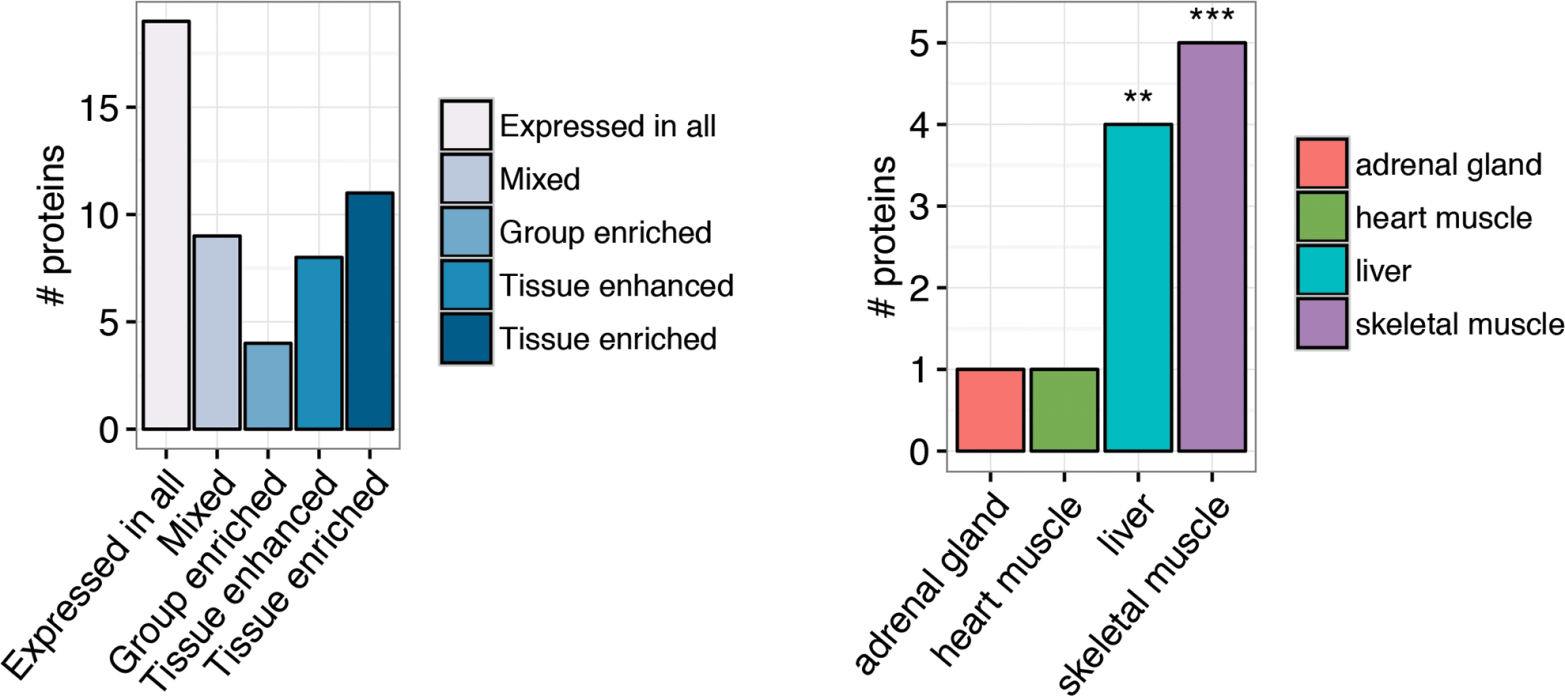
Tissue specificity from Human Protein Atlas. (a) Bar chart showing the number of proteins in each of the categories defined by Human Protein Atlas to classify the proteins according to their level of tissue-specificity. (b) Bar chart showing the tissues in which the tissue-enriched proteins are expressed. The stars above the columns indicate the significance of enrichment analysis (***Fisher’s exact test p-value < 0.0001; ** Fisher’s exact test p-value < 0.001).

To evaluate the biological role of these proteins we first analyzed their tissue-specific expression using the transcript expression levels in Human Protein Atlas (HPA) dataset [19]. This analysis identified eleven proteins of the biomarker (21%) as tissue specific in HPA and, among them, highlighted an over-representation of muscle-specific proteins (Fig 3 and S1 Table). In fact, skeletal muscle resulted a significantly enriched tissue (p-value = 7.831e-05; Fisher’s exact test) with five proteins showing specific expression: Carbonic anhydrase 3 (*CA3*), Creatine kinase M-type (*CKM*), Mitogen-activated protein kinase 12 (*MAPK12*), Tropomyosin beta chain (*TPM2*) and Troponin I, fast-twitch isoform (*TNNI2*). In addition, the biomarker included one protein specific of heart muscle, the Cardiac troponin I (*TNNI3*). Four proteins, despite not being tissue specific, showed elevated expression in a group of tissues—HPA group enriched proteins—that included skeletal or heart muscle (CaMK-II subunit alpha (*CAMK2A*), CaMK-II subunit beta (*CAMK2B*), Heart-type fatty acid-binding protein (*FABP3*) and myoglobin (*MB*)). Notably, the biomarker also showed an over-representation of liver-specific proteins (p-value = 0.006; Fisher’s exact test).

**Fig 4:**
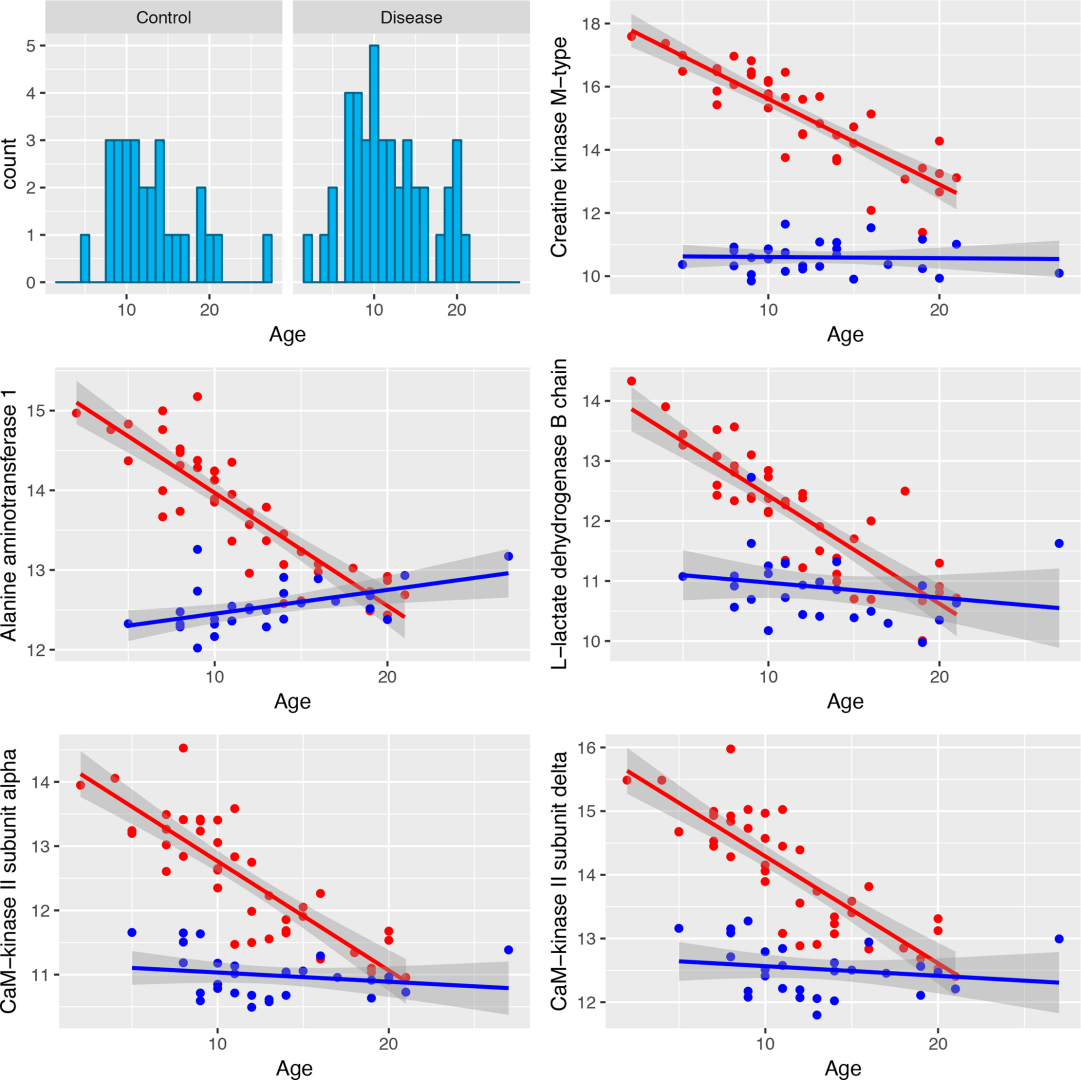
Age-related changes of biomarker levels in DMD patients and control. The first chart shows the age distribution in cases and controls while the other charts show the variation in serum protein level for the five proteins showing the most significant age related change. The red dots correspond to the data for DMD patients while the blue ones to the controls. The lines in the corresponding colors show the regression line, while the gray area corresponds to the confidence interval.

Since the serum protein levels in DMD patients are known to be affected by age, we investigated how the proteins in the biomarker panel change according to the age of patients. After having verified that the age distribution in patients is not significantly different from controls (p-value of Kolmogorov-Smirnov test: 0.82; Fig 4), we tested the presence of a relationship between age and protein levels. We identified 31 proteins whose serum level changes significantly with age. The top 5 proteins are shown in Fig 4 and the full list of biomarker proteins is shown in S2 Fig and S2 Table. With two exceptions, in all cases the protein levels decreased with age in DMD patients while they remain stable in controls. Nine of them showed a significant age-related change also in controls but the variation observed in the two groups was different and did not affect their separation (S3 Fig). These results are not unexpected since the disease is known to affect protein levels along its progression and this change has been related to the progressive loss of muscle mass [20]. Given the presence in the cohort of 28 DMD subjects treated with steroids, we checked if the treatment influenced the protein levels and 32 proteins of the biomarker panel resulted significantly associated with the treatment, indicating proteins potentially affected by the drug (S4 Fig). Despite the presence in the dataset of patients treated with steroids and patients not treated, this did not affect the classification (S5 Fig).

### Gene-set enrichment analysis

To identify the biological processes altered in the disease we first computed the overlap between the 52 biomarker proteins and gene sets retrieved by MSigDB [21]. Specifically, gene set enrichment analysis was performed using hallmark gene sets and the canonical pathways from KEGG, Reactome and Biocarta (Table 3). The analysis with hallmark gene sets confirmed the over-representation of skeletal muscle proteins with a significant enrichment for “myogenesis” gene set (BH corrected p-value 0.000304). The genes coding the biomarker proteins present in the “myogenesis” gene set are: *TNNI2*, *CKM*, *TPM2*, *FABP3*, *MAPK12*, *GSN*, *MB*, *CKB*, *NOTCH1* and *CAMK2B*. In addition to the muscle-related biological functions, Biocarta pathway analysis revealed the presence of proteins involved in the coagulation cascade and pointed out the presence of different signaling pathways differentially regulated in DMD individuals compared with controls (Table 3). In particular, significantly enriched Biocarta pathways included different gene sets related to insulin receptor—PI3K/Akt pathway (igf1-mtor pathway, akt pathway and igf1r pathway), consistent with the already described role of PI3K/Akt in muscle atrophy and hypertrophy [22,23].

**Table 3:** Gene-set enrichment analysis.

pathway	pathway description	# genes	BH adjusted p-value
HALLMARK_MYOGENESIS	Genes involved in development of skeletal muscle (myogenesis)	10	0.000304117
BIOCARTA_AMI_PATHWAY	Acute Myocardial Infarction	5	0.012630235
BIOCARTA_CREB_PATHWAY	Transcription factor CREB and its extracellular signals	5	0.012630235
BIOCARTA_SARS_PATHWAY	The SARS-coronavirus Life Cycle	3	0.023907602
BIOCARTA FIBRINOLYSIS PATHWAY	Fibrinolysis Pathway	4	0.023907602
BIOCARTA CACAM PATHWAY	Ca++/ Calmodulin-dependent Protein Kinase Activation	3	0.028852412
BIOCARTA STATHMIN PATHWAY	Stathmin and breast cancer resistance to antimicrotubule agents	3	0.028852412
BIOCARTA PGC1A PATHWAY	Regulation of PGC-1a	3	0.028852412
BIOCARTA EXTRINSIC PATHWAY	Extrinsic Prothrombin Activation Pathway	4	0.037936344
BIOCARTA BAD PATHWAY	Regulation of BAD phosphorylation	4	0.037936344
BIOCARTA ACH PATHWAY	Role of nicotinic acetylcholine receptors in the regulation of apoptosis	3	0.037936344
BIOCARTA IGF1MTOR PATHWAY	Skeletal muscle hypertrophy is regulated via AKT/mTOR pathway	3	0.046573009
BIOCARTA AKT PATHWAY	AKT Signaling Pathway	3	0.046573009
BIOCARTA INTRINSIC PATHWAY	Intrinsic Prothrombin Activation Pathway	4	0.046573009
BIOCARTA IGF1R PATHWAY	Multiple antiapoptotic pathways from IGF-1R signaling lead to BAD phosphorylation	3	0.046573009
KEGG GLIOMA	Glioma	7	0.01806724
REACTOME UNBLOCKING OF NMDA RECEPTOR GLUTAMATE BINDING AND ACTIVATION	Genes involved in Unblocking of NMDA receptor, glutamate binding and activation	3	0.02196037
REACTOME CREB PHOSPHORYLATION THROUGH THE ACTIVATION OF CAMKII	Genes involved in CREB phosphorylation through the activation of CaMKII	3	0.02196037
REACTOME RAS ACTIVATION UOPN CA2 INFUX THROUGH NMDA RECEPTOR	Genes involved in Ras activation uopn Ca2+ infux through NMDA receptor	3	0.02196037

### Identification of disease sub-networks and molecular regulators characterizing DMD

**Fig 5:**
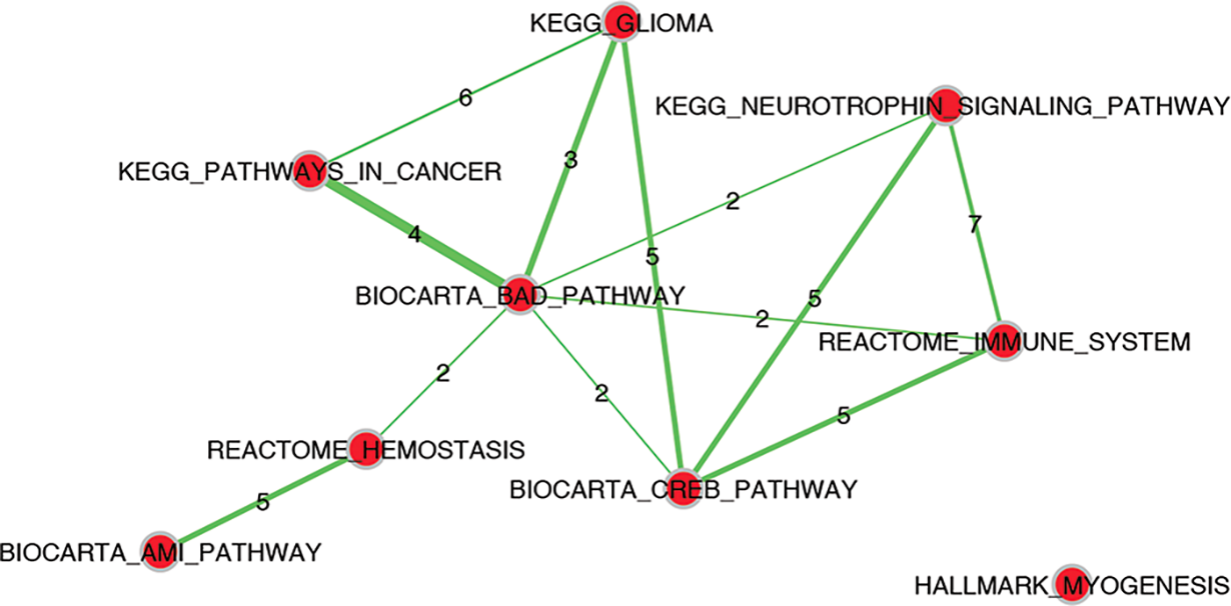
Pathway enrichment map showing the overlap among the pathways identified by NASFinder. The nodes correspond to the pathways and the thickness of the edges connecting them is proportional to the number of shared genes (indicated on the edges).

In the next step of the analysis we further investigated the presence of deregulated regulatory circuits in DMD using a network-based approach. In our workflow we applied NASFinder, a network analysis tool we recently developed [14]. Specifically, we mapped on a global signaling interaction network the 52 proteins in the biomarker panel and we identified the sub-networks connecting these proteins with transcription factors (TF). We opted for the use of TF as source nodes because they are at the top of signaling and regulatory cascades. The connections between TF and proteins of interest were established by NASFinder preferentially through differentially expressed proteins (see details in Materials and Methods). To identify the biological processes associated with the disease regulatory circuits we performed a network-based pathway enrichment analysis. The significant pathways are reported in Table 4 together with the transcription factors that NASFinder identified as the most probable regulators. On the whole, the resulting gene sets are partially overlapping, as shown in Fig 5 and S3 Table, suggesting the presence of a shared regulated biological process that involves multiple pathways. Indeed, only myogenesis did not show genes in common with other pathways and, as a confirmation of its peculiarity, the transcription factor selected as putative regulator was *MEF2C*, a transcription activator specific of muscle genes. In the enrichment map it is interesting to observe the centrality of BioCarta BAD pathway, a gene set related to the regulation of the pro-apoptotic molecule BAD. Among the regulators of the sub-networks the Forkhead family of transcription factors was the most represented with 3 members: *FOXO1*, *FOXO3* and *FOXO4*. They were respectively identified as regulators of the sub-networks enriched in genes involved in acute myocardial infarction pathway, cancer pathways, neurotrophin signaling and immune system pathway. In particular, eleven proteins in the NASFinder FOXO3 sub-network are present in the neurotrophin pathway (p-value = 1.34 x 10^−7^). Seven of them (corresponding to the genes *CAMK2A*, *CAMK2B*, *CAMK2D*, *FASLG*, *MAPK12*, *PIK3CA* and *PIK3R1*) derived from the biomarker while the others (*NFKB1*, *FOXO3*, *MAPK3* and *PIK3CG*) were added by NASFinder on the basis of the differentially expressed proteins. It is interesting to observe that 7 proteins (coded by the genes *MAPK12*, *CAMK2A*, *CAMK2B*, *CAMK2D*, *MAPK3*, *PIK3CA* and *PIK3R1*) were also identified in the Reactome immune system pathway (p-value = 0.003).

**Table 4:** Network-based pathway enrichment analysis.

regulator	# genes in the sub-network	Pathway	# pathway overlapping genes	p-value	Empirical p-value of DMD proximity analysis
TP53	83	KEGG GLIOMA	11	4.82E-10	0.513
FOXO1	89	KEGG PATHWAYS IN CANCER	21	2.57E-09	0.11
FOXO1	89	BIOCARTA AMI PATHWAY	7	8.23E-09	0.086
EGR2	76	BIOCARTA CREB PATHWAY	7	2.55E-08	0.411
FOXO3	74	KEGG NEUROTROPHIN SIGNALING PATHWAY	11	1.73E-07	0.035
MEF2C	98	HALLMARK MYOGENESIS	15	3.38E-07	0.312
MYB	87	REACTOME HEMOSTASIS	20	3.12E-06	0.549
RBPJ	63	BIOCARTA BAD PATHWAY	4	0.0001	0.276
FOXO4	77	REACTOME IMMUNE SYSTEM	21	0.0028	0.481

Considering all the sub-networks and the related pathways pinpointed by NASFinder, PI3-kinase subunit alpha (*PIK3CA*) and PI3-kinase regulatory subunit alpha (*PIK3R1*) were identified as the proteins shared among all pathways, with the exception of myogenesis and myocardial infarction. The two proteins are subunits of phosphatidylinositol 3-kinase, a key element in PI3K/AKT pathway, a regulator of several signaling cascades and in particular of FoxO signaling [24].

**Fig 6:**
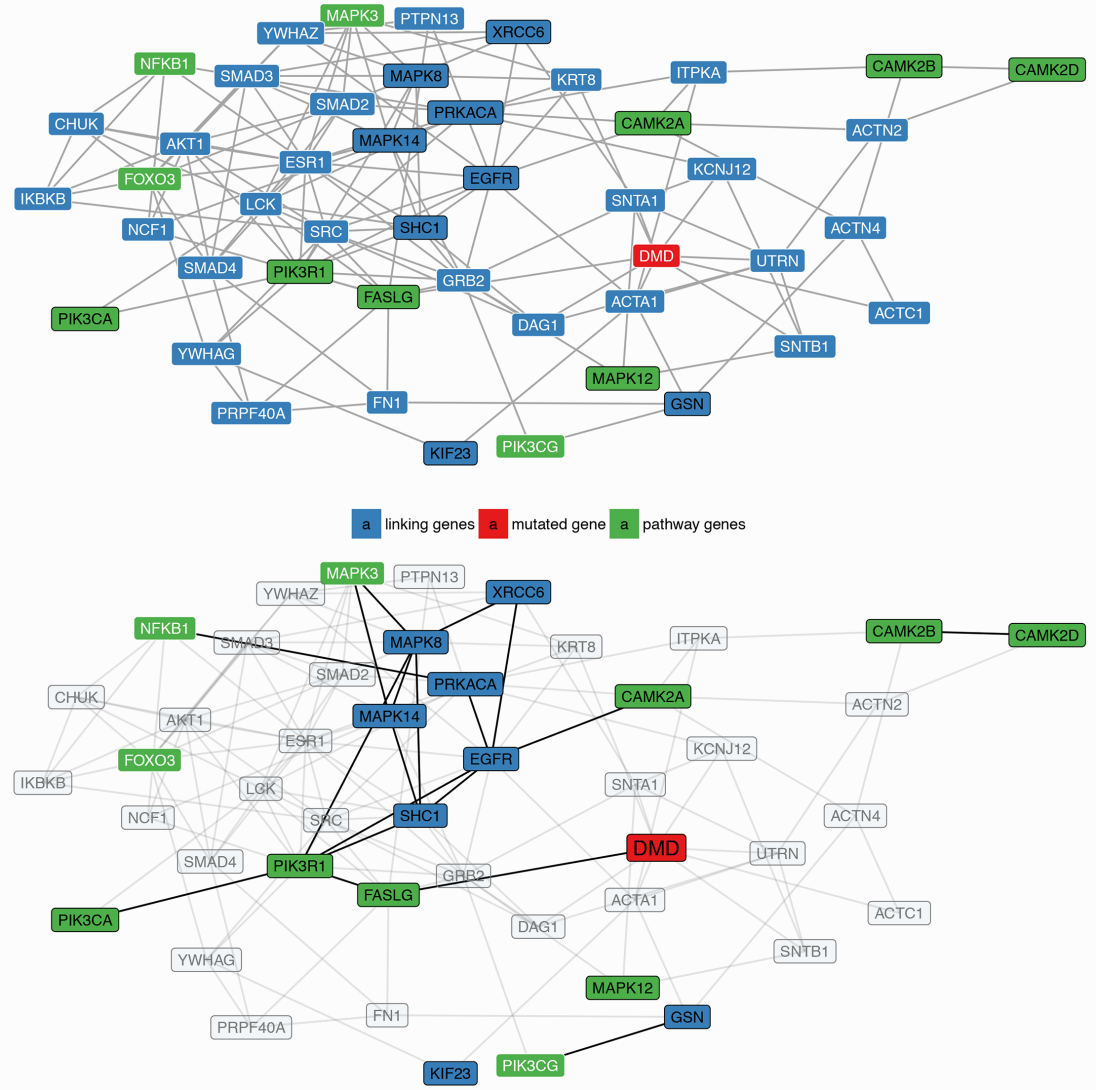
Network-based DMD-pathway proximity analysis. (A) Graphical representation of the subnetwork connecting dystrophin and the proteins in the neurotrophin pathway. The gene symbols corresponding to differentially expressed proteins are written in *black*, those not modulated or not measured with the SomaLogic platform are in *white*. When more than one path with the same shortest distance was identified, only those including differentially expressed genes are shown. (B) *DMD*, the genes in the neurotrophin pathway, the differentially expressed genes and their direct connections are highlighted.

To further investigate the relevance of the identified pathways in the disease biology, we evaluated the closeness in the interactome between the proteins belonging to the pathways described above and dystrophin, the protein mutated in the disease. Expecting that not all the pathway proteins are necessarily involved in the disease, we calculated the shortest path distances between dystrophin and any of the pathway proteins. Among the nine pathways identified using the network-based pathway enrichment analysis (Table 4), the neurotrophin signaling pathway resulted significantly closer to *DMD* than expected by chance (empirical p-value = 0.035; Fig 6). These results offer a network-based support for the involvement of signaling cascades, and specifically PI3K signaling, in the disease.

### Comparison with published studies about proteomics in DMD

We selected five published proteomics studies performed on blood serum of subjects affected by DMD or DMD mouse models [20,25–28] and checked if the proteins in our panel had been already described as DMD biomarkers (S4 Table). Twenty-seven of the 52 proteins included in our long biomarker panel were already identified as differentially present between serum of DMD cases compared with controls in a previous analysis performed by Hathout and collaborators [20]. To further assess the similarity of the results between the two studies, we investigated their protein signature with our analysis workflow. Using Gene Set Enrichment Analysis, although none was significant after multiple test correction, some of the most enriched pathways are shared between the two studies (see S5 Table). Using NASFinder, the analysis identified 12 statistically significant pathways (S6 Table). Interestingly, three of them (immune system, hemostasis and myogenesis) are shared with our analysis, and for myogenesis the transcription factor identified as a close regulator is the same. Comparing our results with the study by Ayoglu and collaborators [25], which was performed with an antibody bead array platform, creatine kinase and carbonic anidrase were the proteins in common with those of our long panel. *GSN* was already pinpointed in mass spectrometry analysis of human serum by Cynthia Martin et al. [26]. In the comparison we also included two studies performed using a DMD mouse model. In this case, nine proteins were in common with a study that used the SOMAscan technology for proteomic profiling [27] and five proteins with a study based on mass spectrometry [28].

### Evaluation of gene expression patterns in DMD skeletal muscle

The serum proteomics detects the proteins present in the blood, however they can derive from muscle degenerating fibers released into the systemic circulation rather than reflecting expression regulation. Based on this consideration we decided to analyze a type of evidence complementary to our proteomics data; specifically we selected a public dataset of genome-wide expression data obtained from the skeletal muscle of DMD and controls (GSE1004). This dataset contains expression data from quadriceps of patients with clinical symptoms consistent with a DMD diagnosis, whose biopsies were shown to be dystrophin deficient [29]. After having calculated the differentially expressed genes, to investigate the biological processes affected, pathway enrichment analysis was performed using the differentially expressed genes as input and we evaluated the overlap with the pathways identified by NASFinder. With the exception of two Biocarta gene sets, all the pathways identified by NASFinder from the proteome datasets resulted significantly enriched, as reported in Table 5. We further evaluated the dysregulation of these pathways in a dataset of DMD patients in early phases of the disease and controls (GDS3027). These data allowed us to test the dysregulation of the pathways in a pre-symptomatic phase of the disease. With the exception of Biocarta gene sets, the other pathways resulted among the top 20 results (S7 Table).

**Table 5:** Comparison of NASFinder results with pathway enrichment analysis of dataset GSE1004.

Pathway	# Genes in Overlap	FDR qvalue
HALLMARK MYOGENESIS	31	7.03E-24
REACTOME IMMUNE SYSTEM	50	6.71E-16
REACTOME HEMOSTASIS	31	2.67E-12
KEGG PATHWAYS IN CANCER	22	2.83E-09
KEGG GLIOMA	7	0.000083
KEGG NEUROTROPHIN SIGNALING PATHWAY	8	0.000647
BIOCARTA AMI PATHWAY	3	0.0195
BIOCARTA CREB PATHWAY	NA	NA
BIOCARTA BAD PATHWAY	NA	NA

## Discussion

In this study we adopted a system biology approach to study Duchenne muscular dystrophy. Our workflow is based on the combination of two complementary methodologies: biomarker identification and network analysis. We applied the algorithm for biomarker identification to point out the essential features of the biological system, i.e. the minimum set of proteins able to fully separate healthy from affected subjects, and then we applied the network analysis to unravel the complex interactions among the proteins. Taking advantage of the flexibility of our algorithm, we calculated two different protein lists, a short one and a long one. All the proteins present in the short biomarker panel have been already described as DMD biomarkers in Hathout et al. [20] and they are related to the main pathological features observed in DMD [6]. However, the short biomarker panel described here, compared with the signature in Hathout et al. [20], has the advantage of being able to classify the subjects with only six proteins achieving perfect accuracy and high statistical significance (permutation test p-value < 0.001). Our long biomarker panel has size comparable to the disease signature reported in Hathout et al. (52 vs 46 proteins, respectively) but only approximately half of the proteins (26) are in common between the two lists. Such low overlap is not surprising in view of the fact that the different approaches used in the two studies (classification-oriented vs statistical significance) have been shown not to be equivalent [30]. From an investigational point of view, our classification-oriented list provides some additional insights on less known aspects of the disease pathophysiology and in particular to identify deregulated pathways and potential regulators. Indeed, in addition to proteins with a muscle origin, likely found in the serum proteome as a consequence of sarcolemma leakage [31], our analysis pointed out several proteins involved in cell signaling that, given their biological role, it is not surprising to detect in blood serum. Our results are in line with growing evidences suggesting that the cellular structural instability caused by the lack of dystrophin affects different signaling pathways [32–34]. In particular, the network-based pathway enrichment analysis implemented in NASFinder identified nine pathways as significantly enriched and, among the influential nodes, different transcription factors belonging to the FoxO family were identified as possible regulators. In humans FoxO proteins are all expressed in skeletal muscle and they have been implicated in muscle homeostasis [35]. Specifically, regarding muscle mass, FoxO proteins were shown to enhance proteolysis through the ubiquitin-proteasome and the autophagy-lysosome system [36]. Notably, a study on sarcopenia, the physiological age-related loss of muscle mass, identified *FOXO3* as an up-regulated gene in muscles from old subjects [37] and another study found increased *FOXO1* mRNA in aged muscle [38]. However, findings from a more recent study suggest that sarcopenia is not due to FoxO activation [39], highlighting the need for further studies to better understand the role of FoxO proteins in physiological and pathological muscle conditions. Among the sub-networks regulated by FoxO proteins, the neurotrophin signaling pathway, a cascade of biological processes regulating cell survival and apoptosis [40], was identified as enriched in our analysis. Although neurotrophins have been mainly linked to the nervous system [41], recent studies indicated their involvement in skeletal muscle adaptation and regeneration [42]. Specifically, experimental evidences showed that neurotrophins are involved in muscle regeneration in dystrophic mice [43] and the nerve growth factor, the paradigm of neurotrophin family, was identified in muscles from patients affected by DMD [44]. Interestingly, a recent study performed using the *mdx* mouse, a model of DMD, linked apoptosis of myofibers to the presence of connexin, a protein channel involved in NF-κB activation, iNOS expression and apoptotic cell death [45]. In our analysis *NFKB1*, a DNA binding subunit of the NFKB protein complex, is present in the FOXO3 sub-network defined by NASFinder and the protein is part of the neurotrophin-related signaling. The network proximity analysis (Fig 6) suggested that the dysregulation of the neurotrophin signaling pathway in patients affected by DMD might be mediated by Fas ligand (*FASLG)*, a mediator of the cellular apoptotic signal that is a direct interactor of the dystrophin protein [46]. A link between Duchenne muscular dystrophy and *FASLG* has already been reported by Abdel-Salam et al. [47], which observed significantly increased levels of FasLG mRNA expression in blood of DMD patients compared to controls. In addition to neurotrophin signaling, our analysis pointed out, among the FoxO-related sub-networks, an enrichment for the immune system signaling with FOXO4 identified as a regulator. The role of FoxO proteins in immune system has been already described [48] and interestingly, this process, together with hemostasis, another significant pathway in NASFinder analysis, is a key player in fibrosis, a prominent feature of dystrophic muscle [49]. It is thus conceivable that the dysregulation of blood proteins belonging to these pathways is a consequence of the fibrotic process ongoing in muscles of DMD patients. Notably, modulation of the immune response was suggested as a potential means to alleviate the disease intensity [50]. The other FoxO transcription factor with a pathway enriched sub-network was FOXO1. In this case two pathways resulted enriched: BioCarta acute myocardial infarction and KEGG pathways in cancer. While the presence of cardiac dysfunctions in subjects affected by DMD is common [51], we consider the presence of enrichment in cancer pathways (also KEGG glioma in *TP53* network resulted enriched) a consequence of the bias introduced by the high number of genes annotated in cancer pathways. Indeed, we noticed that most of the genes in cancer pathways also belong to other enriched gene sets (S3 Table).

In conclusion, in this study we presented a new bioinformatics workflow based on biomarker identification and network analysis that we used to extract biological insights from proteomics data obtained from a cohort of DMD subjects and controls. This methodology allowed us to identify different pathways deregulated in the disease and to pinpoint a putative role of FoxO signaling in DMD. One limitation of this study is the lack of validation of the biomarker panel classification performance using a second independent cohort of subjects. To partially address this issue and avoid overfitting, we trained the algorithm that computed the biomarkers according to a 5-fold cross-validation scheme. We also used a second dataset of a very different nature (transcriptional profile of muscle from an unrelated cohort) to confirm the end results of our analysis and we obtained a substantial confirmation of the results. Another limitation of this study, as any form of *in silico* analysis, is that it relies entirely on observational data. Therefore, our results will have to be validated by means of functional studies. Despite these limitations, our workflow derives its strength from the convergence of multiple types of evidences from independent experiments, crossed with existing knowledge about the biology of the disease.

## Materials and methods

### Dataset

The dataset analyzed in this study was obtained from a collaboration with SomaLogic and includes subjects previously analyzed by Hathout and coworkers [20]. The present dataset consists of 42 subjects affected by DMD and 28 healthy, age-matched volunteers (PPMD-C cohort). The group of patients included 28 individuals treated with steroids. For each subject a proteomic profile was obtained using the SOMAscan technology by SomaLogic. Briefly, this high throughput method is based on the use of aptamers with high affinity for the proteins. The presence in the aptamers of a DNA sequence allows the quantification of the protein levels in a simple way using microarray-based technology. Further details about the cohort and the proteomic assay can be obtained from [20]. The original study was approved by the Cincinnati Children’s Hospital Medical Center Institutional Review Board and informed consent was obtained from patients or their parents or legal guardians. All methods were performed in accordance with relevant guidelines and regulations.

### Biomarkers identification

Protein biomarkers have been identified by means of an enhanced version of the rank-based classification method previously introduced in [12,13]. Briefly, after a preliminary protein selection phase based on the Wilcoxon test, the classification method ranks the filtered proteins by expressions level separately for each sample and then it produces a set of subject-specific signatures, where each signature is the list of the first n1 and the last n2 proteins in the ranking (n1 and n2 have the same value for all subjects and they are parameters estimated by the method). An all-to-all signature comparison is then carried out using a distance metric based on a weighted enrichment score [52], resulting in a distance matrix that systematically quantifies the degree of similarity between the subjects. Subjects are then classified by the algorithm into the two groups of controls and diseased, by assigning each sample to the group of subjects whose elements have the lowest averaged distance from the sample. Finally, a protein biomarker is extracted, which collects all the proteins included in at least one subject-specific signature. Therefore, the proteins included in a biomarker are those required to compute the corresponding subject classification.

In the enhanced version used here, the original classification method has been extended as in our previous studies [53,54] with a genetic optimizer that automatically selects the method parameters (signature length and feature selection stringency) to find the best compromise between biomarker length and classification accuracy. In particular, the two biomarkers herein presented provide the shortest and the longest protein list allowing classification accuracy close to 100%. In the case of the longest biomarker, protein levels have also been preprocessed to emphasize expression fold-changes by dividing protein levels by their averaged value in the dataset.

To avoid overfitting, in all the considered cases the algorithm has been trained according to a 5-fold cross-validation scheme, where 20% of the subjects were set aside for validation at each round, and the remaining ones are used as training set for determining the biomarker. Moreover, we also evaluated the statistical significance of the analysis by means of a permutation test in which we compared the observed classification accuracy of the method with an empirical distribution of accuracy values obtained by 1000 random permutations of the protein labels. As a result of the permutation test we obtained in all cases a p-value < 0.001.

Linear regression was run to identify proteins in the biomarker panel associated with age, after having log2 scaled the data. Furthermore, the presence of a treatment effect on protein levels was tested using Wilcoxon test. For both analyses, p-values were adjusted for multiple test correction using the Benjamini-Hochberg procedure.

### Tissue specificity and gene set enrichment analysis

Tissue specificity was evaluated using the transcriptomic data in the Human Protein Atlas (http://www.proteinatlas.org/) [19]. In this database proteins are classified as tissue enriched (mRNA levels in one tissue at least five times higher than all other tissues), group enriched (mRNA levels in a group of 2 to 7 tissues at least five times higher than all other tissues), tissue enhanced (mRNA levels in a particular tissue at least five times the average level in all tissues), expressed in all (mRNA detected in all tissues), mixed (detected in a subset of tissues and expression not markedly elevated in any) and not detected. To evaluate the enrichment in a particular tissue we performed a Fisher’s exact test comparing the tissue enriched proteins in the biomarker panel with the entire SomaLogic panel.

To perform gene set enrichment analysis we downloaded the gene sets from the Molecular Signatures Database website [21], v5.1, updated in January 2016. In particular, the following gene set collections were investigated: hallmark, BioCarta (http://www.biocarta.com/), KEGG[55] and Reactome [56]. Hallmark is a refined collection defined by MSigDB derived from multiple founders to point out relevant information about biological conditions avoiding redundancy [57]. The enrichment was evaluated using the SomaLogic panel as a background and the statistical significance was tested using the hypergeometric distribution (phyper function implemented in the R software).

### Network analysis

To explore the interactions among the proteins in the biomarker panel and identifying putative regulators, we applied NASFinder, a tool we recently developed to analyze the connections among genes of interest and their upstream regulators [14]. Briefly, the proteins in the biomarker panel are mapped to a reference protein-protein interaction network, that is, the human signaling network from the Wang Lab - http://www.cancer-systemsbiology.org/dataandsoftware.htm. Then, the subnetwork connecting the proteins of interest, in our case the proteins in the biomarker panel, and the closest transcriptional factor (molecules selected as regulators) is defined. If a protein of interest is not present in the reference network it is added, if possible, from BioGRID [58] as detailed in [14]. The paths that connect transcription factors and biomarker proteins are established by NASFinder preferentially through differentially expressed proteins and they were identified in our study using the Bioconductor package limma. Specifically, proteins were selected as significant when they showed a Benjamini-Hochberg adjusted p-value less than 0.05 in the comparison between DMD subjects and controls. Once the subnetworks were identified, we evaluated their overlap with reference pathways from KEGG, Reactome, Biocarta and the hallmark collection in MSigDB, as in the gene-set enrichment analysis. The significance of the overlap was calculated using one-sided Fisher's exact test. The degree of overlap among the pathways identified by NASFinder was visualized using the Cytoscape App Enrichment Map v2.1.0 [59], run using Cytoscape 3.4.0. Enrichment Map was run using default parameters and Overlap coefficient as a measure of similarity.

The network-based proximity analysis between *DMD* and the pathway genes was performed with the R package igraph, using the protein-protein interaction network from the Human Protein Reference Database (HPRD) [60]. This background network was preferred to the signaling network used for the identification of pathways and regulators because it includes only physical interactions among proteins instead of regulatory interactions. For all the pathways, all the shortest paths from *DMD* to the genes were calculated and the shortest path distance was defined as the minimal distance found. To assess the significance, we selected 1000 random genes and, for each of them, we calculated the minimal shortest distance to the pathway. We thus obtained an empirical distribution that was used to calculate the proportion of samples with a minimal shortest distance less or equal to the distance obtained using *DMD*.

### Gene expression analysis

The datasets GSE1004 and GDS3027 were downloaded from NCBI GEO using the R Bioconductor package GEOquery. Dataset GSE1004 was obtained measuring RNA expression in quadriceps biopsies and it includes 12 DMD patients and 12 controls. The DMD biopsies were from young (5- to 7-year-old) males showing clinical symptoms consistent with a DMD diagnosis, and the biopsies were shown to be dystrophin deficient by immunofluorescence and/or Western blotting [29]. For the purpose of this study we analyzed the data from the platform HG_U95Av2 (GPL8300) which includes 11 controls and all the 12 cases. Dataset GDS3027 includes the expression profiles of skeletal muscles from 23 children in a pre-symptomatic phase of Duchenne muscular dystrophy and 14 controls [61]. The analysis of differentially expressed genes was performed with the R Bioconductor package limma. The gene-set enrichment analysis was performed using the MSigDB tool of the GSEA package (http://www.broadinstitute.org/gsea/msigdb/index.jsp) querying the same gene sets collections used in the network analysis (obtained from hallmark, KEGG, Biocarta and Reactome).

## Supporting information

S1 Table Tissue-specificity of proteins in the long biomarker panel. The data were retrieved from Human Protein Atlas.
(PDF)

S2 Table Association with age in disease. For all the proteins in the long biomarker panel the results of the association analysis with age are shown.
(PDF)

S3 Table NASFinder results. For each pathway that resulted overrepresented the genes in the corresponding sub-network are reported.
(PDF)

S4 Table Literature comparison. For all the proteins in the long biomarker panel the result of a literature search about DMD proteomics studies identifying the same protein is reported.
(PDF)

S5 Table Gene-set enrichment analysis using the protein signature in Hathout et al. (PDF)

S6 Table NASFinder results obtained using the protein signature in Hathout et al. (PDF)

S7 Table Pathway enrichment analysis of significantly enriched genes obtained from the transcriptomic dataset GDS3027. The top 20 pathways for each database are shown.
(PDF)

S1 Fig Principal component analysis of a subset of the DMD dataset that includes only the 6 proteins in the short biomarker panel. (PDF)

S2 Fig Age-related protein level variation in DMD subjects. The plots show all the proteins in the long biomarker panel showing a significant correlation with age.
(PDF)

S3 Fig Charts showing proteins with a significant association with age in both cases and controls. (PDF)

S4 Fig Charts showing the treatment effect in patients. Only the proteins in the long biomarker panel with a significant difference are shown.
(PDF)

S5 Fig Network plots showing the case control differentiation using the proteins in the biomarker panels. The nodes of the network represent the samples, while colors indicate their status (controls, disease without treatment, diseases with treatment). The length of the edges is proportional to level of similarity between the sample signatures. In both cases we can observe that controls and disease samples cluster together forming two separate groups, while this does not happen if we consider the treatment (red nodes and orange ones are not clearly separated). (A) Short biomarker panel. (B) Long biomarker panel.
(PDF)
