# Supplementary material for: Combined use of protein biomarkers and network analysis unveils deregulated regulatory circuits in Duchenne muscular dystrophy
Source: PLoS One. 2018 Mar 12;13(3):e0194225. doi: 10.1371/journal.pone.0194225 (PMC5846794; doi:10.1371/journal.pone.0194225)
Supplement: S1 Table — The data were retrieved from Human Protein Atlas. (PDF) [file pone.0194225.s001.pdf]

**Table S1.** Tissue-specificity of proteins in the long biomarker panel. The data were retrieved from Human Protein Atlas

| **Entrez Gene Symbol** | **Subcellular location** | **RNA tissue category** | **RNA TS FPKM** | **FPKM max in non specific** |
|---|---|---|---|---|
| ALCAM | Cytoplasm | Mixed | | lung: 70.9 |
| ANP32B | | Expressed in all | | tonsil: 112.1 |
| CA3 | | Tissue enriched | skeletal muscle: 1438.4 | prostate: 107.8 |
| CADM1 | Cell Junctions | Mixed | | lung: 63.4 |
| CAMK2A | | Group enriched | cerebral cortex: 134.7;skeletal muscle: 69.2 | heart muscle: 8.8 |
| CAMK2B | | Group enriched | cerebral cortex: 99.4;heart muscle: 47.0;skeletal muscle: 44.9 | adrenal gland: 10.5 |
| CAMK2D | | Expressed in all | | heart muscle: 129.3 |
| CD109 | | Mixed | | skin: 16.1 |
| CD200R1 | | Mixed | | spleen: 8.4 |
| CD55 | Nucleus | Expressed in all | | lung: 222.3 |
| CD86 | | Mixed | | appendix: 35.3 |
| CDH5 | | Tissue enhanced | placenta: 137.9 | lung: 84.1 |
| CKB | | Expressed in all | | prostate: 589.1 |
| CKM | | Tissue enriched | skeletal muscle: 8139.3 | heart muscle: 1172.1 |
| CX3CL1 | Plasma membrane | Mixed | | lung: 54.3 |
| DNAJC19 | | Expressed in all | | kidney: 35.6 |
| EGFR | Plasma membrane, Cytoplasm | Tissue enhanced | placenta: 91.3 | skin: 43.2 |
| ENG | Nucleus but not nucleoli, Cytoplasm | Expressed in all | | heart muscle: 127.7 |
| FABP3 | Golgi apparatus | Group enriched | heart muscle: 2098.6;skeletal muscle: 459.8 | kidney: 135.5 |
| FAP | | Tissue enhanced | endometrium: 37.1 | smooth muscle: 28.3 |
| FASLG | | Tissue enhanced | lymph node: 4.9 | spleen: 3.1 |
| FGA | | Tissue enriched | liver: 4181.2 | stomach: 28.1 |
| FGB | | Tissue enriched | liver: 4244.6 | kidney: 23.5 |
| FGG | | Tissue enriched | liver: 3876.1 | lung: 30.0 |
| GDF11 | | Mixed | | cerebral cortex: 7.9 |
| GPI | Plasma membrane, Cytoplasm, Nucleus but not nucleoli | Expressed in all | | heart muscle: 256.2 |
| GPT | | Tissue enhanced | liver: 64.6 | skeletal muscle: 35.0 |
| GSN | Cytoskeleton (Actin filaments) | Expressed in all | | adipose tissue: 776.7 |

| | | | | |
|---|---|---|---|---|
| HDGFRP2 | Nucleus but not nucleoli | Expressed in all | | testis: 37.1 |
| IGF1R | Vesicles | Expressed in all | | fallopian tube: 18.8 |
| IL18BP | Nuclear membrane | Expressed in all | | spleen: 49.8 |
| JAG1 | Plasma membrane, Golgi apparatus | Expressed in all | | placenta: 42.0 |
| KIT | | Mixed | | thyroid gland: 46.5 |
| LDHB | Cytoplasm | Expressed in all | | heart muscle: 1123.5 |
| MAPK12 | Nucleus but not nucleoli, Cytoplasm | Tissue enriched | skeletal muscle: 107.8 | cerebral cortex: 13.0 |
| MB | | Group enriched | heart muscle: 3937.4;skeletal muscle: 5728.1 | esophagus: 467.7 |
| MDH1 | Centrosome, Cytoplasm | Expressed in all | | heart muscle: 872.8 |
| NOTCH1 | | Expressed in all | | skin: 16.4 |
| PIK3CA | Cytoplasm | Expressed in all | | adipose tissue: 18.0 |
| PIK3R1 | Cytoplasm | Expressed in all | | fallopian tube: 83.8 |
| PLA2G2A | | Tissue enhanced | rectum: 651.2;small intestine: 869.9 | colon: 566.9 |
| PLAT | Cytoskeleton (Actin filaments) | Mixed | | urinary bladder: 122.2 |
| PROC | | Tissue enriched | liver: 170.7 | kidney: 18.9 |
| PSPN | | Not detected | | fallopian tube: 0.9 |
| RELT | Nucleus but not nucleoli | Tissue enhanced | bone marrow: 61.4 | spleen: 14.1 |
| RET | Plasma membrane, Cytoplasm, Vesicles | Tissue enriched | adrenal gland: 18.7 | salivary gland: 3.6 |
| RPS7 | Cytoplasm, Nucleus | Expressed in all | | ovary: 120.4 |
| SEMA6A | | Expressed in all | | placenta: 39.9 |
| TNFRSF17 | | Tissue enhanced | tonsil: 41.8 | lymph node: 26.1 |
| TPM2 | | Tissue enriched | skeletal muscle: 6561.6 | smooth muscle: 981.0 |
| TNNI2 | | Tissue enriched | skeletal muscle: 2919.8 | esophagus: 101.6 |
| TNNI3 | | Tissue enriched | heart muscle: 2157.7 | skeletal muscle: 6.6 |
