# Supplementary material for: Combined use of protein biomarkers and network analysis unveils deregulated regulatory circuits in Duchenne muscular dystrophy
Source: PLoS One. 2018 Mar 12;13(3):e0194225. doi: 10.1371/journal.pone.0194225 (PMC5846794; doi:10.1371/journal.pone.0194225)
Supplement: S2 Table — For all the proteins in the long biomarker panel the results of the association analysis with age are shown. (PDF) [file pone.0194225.s002.pdf]

Table S2. Association with age in disease. For all the proteins in the long biomarker panel the results of the association analysis with age are shown.

somalogicID	UniProt ID	beta	95% CI	p-value	BH adjusted p-value
2212-69 1	P00750	0.00592591	-0.01 - 0.02	0.490943781	0.554979926
2475-1 3	P10721	0.019586947	-0.01 - 0.05	0.195151004	0.263959597
2665-26 2	Q02223	0.019505418	-0.01 - 0.05	0.184432396	0.259202286
2670-67 4	P06732	-0.271044409	-0.32 - -0.22	8.04E-14	2.09E-12
2677-1 1	P00533	-0.008898478	-0.02 - 0	0.197969698	0.263959597
2692-74 2	P14555	-0.041494073	-0.07 - -0.02	0.001323199	0.002752254
2696-87 2	O60542	0.035368415	0.02 - 0.05	3.65E-05	9.04E-05
2765-4 3	O95390	-0.032037144	-0.05 - -0.02	0.000322126	0.00069794
2796-62 2	P02671<br>P02675<br>P02679	-0.114518085	-0.14 - -0.09	1.77E-12	1.53E-11
2819-23 2	P33151	-0.008385133	-0.03 - 0.01	0.427218744	0.493674993
2827-23 2	P78423	0.010143189	-0.01 - 0.03	0.214908063	0.279380482
2961-1 2	P04070	-0.005874716	-0.02 - 0.01	0.405623099	0.490520957
3042-7 2	P02144	-0.217872215	-0.27 - -0.17	1.22E-10	5.29E-10
3052-8 2	P48023	0.0013531	-0.02 - 0.02	0.895872861	0.931707775
3073-51 2	O95998	0.020710804	0 - 0.05	0.101651249	0.151024713
3220-40 2	P07949	-0.001761371	-0.02 - 0.02	0.841590236	0.907060691
3290-50 2	Q6YHK3	-0.016176836	-0.04 - 0	0.100895987	0.151024713
3326-58 2	Q9BY67	-0.01906117	-0.03 - 0	0.015829787	0.029398176
3350-53 2	Q9UQM7	-0.169821224	-0.2 - -0.14	6.43E-13	8.35E-12
3351-1 1	Q13554	-0.16601308	-0.2 - -0.13	2.85E-12	2.12E-11
3390-72 2	P42336<br>P27986	-0.038556529	-0.05 - -0.02	1.23E-06	3.36E-06
3419-49 2	Q13557	-0.167948601	-0.2 - -0.13	1.45E-12	1.51E-11
3709-4 2	P24298	-0.141795372	-0.17 - -0.12	4.79E-14	2.09E-12
3714-49 2	P12277<br>P06732	-0.24070904	-0.29 - -0.19	4.64E-12	2.91E-11
3799-11 2	P07451	-0.174228926	-0.21 - -0.13	5.15E-11	2.68E-10
3853-56 1	P40925	-0.137400423	-0.19 - -0.09	2.34E-06	6.08E-06
3864-5 2	P62081	-0.039990492	-0.07 - -0.01	0.002756042	0.005307933
3890-8 2	P07195	-0.180194502	-0.21 - -0.15	3.09E-13	5.36E-12
4179-57 3	None	-0.139081504	-0.17 - -0.11	6.80E-11	3.21E-10
4194-26 3	Q92688	-0.103240878	-0.13 - -0.07	7.12E-09	2.65E-08
4232-19 2	P08069	0.005811017	-0.01 - 0.02	0.426417853	0.493674993
4272-46 2	P06744	-0.17936364	-0.23 - -0.13	1.81E-09	7.26E-09
4472-5 2	P07951	-0.035088943	-0.05 - -0.02	0.000111055	0.000262493
4545-53 3	Q96DA6	-0.008426035	-0.01 - 0	0.001798259	0.003596518
4553-65 3	Q7Z4V5	-0.004742399	-0.01 - 0	0.061088871	0.096261251
4696-2 2	P05413	-0.159821307	-0.21 - -0.11	1.19E-08	3.86E-08
4775-34 3	P06396	-0.01707174	-0.03 - 0	0.028658223	0.048071857
4907-56 1	P02671<br>P02675	-0.084408669	-0.11 - -0.06	5.37E-08	1.55E-07

	P02679				
4908-6 1	P17813	0.004098797	-0.01 - 0.02	0.660989501	0.731307533
4989-7 1	P02679	-0.103815798	-0.13 - -0.07	2.15E-08	6.58E-08
5005-4 1	P53778	-0.162143993	-0.2 - -0.13	5.04E-12	2.91E-11
5029-3 1	Q12884	-0.039854512	-0.06 - -0.02	0.000274953	0.000621632
5069-9 3	P08174	-0.000403626	-0.02 - 0.02	0.963563838	0.982457247
5092-51 3	P78504	0.013331932	0 - 0.03	0.056702156	0.092141004
5103-30 3	Q8TD46	0.005680319	-0.01 - 0.02	0.327226479	0.405137545
5107-7 2	P46531	-0.008233286	-0.02 - 0.01	0.229024104	0.290469596
5115-31 3	Q969Z4	-0.000962443	-0.01 - 0.01	0.854730267	0.907060691
5122-92 2	Q9H2E6	0.019390314	0 - 0.04	0.023930753	0.041479972
5337-64 3	P42081	0.000176201	-0.02 - 0.02	0.982558427	0.982558427
5440-26 3	P48788	-0.146286043	-0.19 - -0.11	7.81E-09	2.71E-08
5441-67 3	P19429	-0.035135993	-0.06 - -0.01	0.017318128	0.031053195
5451-1 3	Q13740	-0.011415572	-0.03 - 0.01	0.183925477	0.259202286
