# Supplementary material for: Combined use of protein biomarkers and network analysis unveils deregulated regulatory circuits in Duchenne muscular dystrophy
Source: PLoS One. 2018 Mar 12;13(3):e0194225. doi: 10.1371/journal.pone.0194225 (PMC5846794; doi:10.1371/journal.pone.0194225)
Supplement: S3 Table — For each pathway that resulted overrepresented the genes in the corresponding sub-network are reported. (PDF) [file pone.0194225.s003.pdf]

Table S3. NASFinder results. For each pathway that resulted overrepresented the genes in the corresponding sub-network are reported.

KEGG PATHWAYS IN CANCER	KEGG GLIOMA	KEGG NEUROTROPHIN SIGNALING PATHWAY	REACTOME IMMUNE SYSTEM	REACTOME HEMOSTASIS	HALLMARK MYOGENESIS	BIOCARTA AMI PATHWAY	BIOCARTA CREB PATHWAY	BIOCARTA BAD PATHWAY
JUN	IGF1R	NFKB1	PTK2B	CDK2	TNNI2	PROS1	CAMK2A	IGF1R
STAT3	MDM2	MAPK12	IFNG	SH2B2	CKM	FGG	CAMK2B	KIT
IGF1R	EGFR	CAMK2A	IL1B	EP300	TPM2	PROC	CAMK2D	PIK3CA
LAMC2	PTEN	CAMK2B	IRF6	F2	FABP3	FGA	AKT1	PIK3R1
ARNT	CAMK2A	CAMK2D	MEF2C	FGA	MAPK12	PLG	PIK3CA	
TRAF2	CAMK2B	FOXO3	FOXO4	FGB	MEF2C	FGB	PIK3R1	
FOXO1	CAMK2D	MAPK3	PIK3CA	FGG	GSN	PLAT	MAPK1	
PLCG2	TP53	PIK3CA	PIK3R1	GATA3	MEF2D			
KIT	PIK3CA	FASLG	PPP2R1A	HSPA5	SORBS1			
SMAD2	PLCG2	PIK3CG	MAPK3	ARRB1	FGF2			
ERBB2	PIK3R1	PIK3R1	MAPK8	MYB	MEF2A			
RXRA			MAPK12	NRAS	CKB			
RET			STAT3	PIK3CA	PSEN2			
EGFR			BTK	PIK3R1	NOTCH1			
AR			UBE2A	PLAT	CAMK2B			
TP53			SUMO1	PPP2R5D				
PIK3CA			CAMK2A	PROC				
KLK3			CAMK2B	RAP1A				
FASLG			CAMK2D	TP53				
IL6			CD28	YES1				
PIK3R1			CD86					
