# Supplementary material for: Combined use of protein biomarkers and network analysis unveils deregulated regulatory circuits in Duchenne muscular dystrophy
Source: PLoS One. 2018 Mar 12;13(3):e0194225. doi: 10.1371/journal.pone.0194225 (PMC5846794; doi:10.1371/journal.pone.0194225)
Supplement: S4 Table — For all the proteins in the long biomarker panel the result of a literature search about DMD proteomics studies identifying the same protein is reported. (PDF) [file pone.0194225.s004.pdf]

Table S4. Literature comparison. For all the proteins in the long biomarker panel the result of a literature search about DMD proteomics studies identifying the same protein is reported.

SYMBOL	Hathout et al, 2015	Coenen-Stass et al,2015	Ayoglu et al, 2014	Hathout et al, 2014	Cynthia Martin et al, 2014
CA3	yes	-	yes	-	-
MAPK12	yes	-	-	-	-
TNNI2	yes	yes	-	-	-
CKM	yes	-	yes	yes	-
CKB	-	-	yes	-	-
RET	yes	-	-	-	-
GPI	yes	-	-	-	-
MB	yes	yes	-	yes	-
CAMK2A	yes	yes	-	-	-
FABP3	yes	yes	-	yes	-
TNNI3	yes	yes	-	-	-
CDH5	yes	-	-	-	-
ANP32B	yes	yes	-	-	-
GPT	yes	-	-	-	-
FGG	yes	-	-	yes	-
FGA	yes	-	-	-	-
FGB	yes	-	-	-	-
CAMK2D	-	yes	-	-	-
CD55	yes	-	-	-	-
RPS7	yes	-	-	-	-
PIK3CA	-	-	-	-	-
PIK3R1	-	-	-	-	-
LDHB	yes	yes	-	-	-
RELT	yes	-	-	-	-
ENG	-	-	-	-	-
CAMK2B	-	yes	-	-	-
GSN	yes	-	-	-	yes
CX3CL1	-	-	-	-	-
PLA2G2A	yes	-	-	-	-
DNAJC19	-	-	-	-	-
JAG1	yes	-	-	-	-

PLAT	-	-	-	-	-
KIT	-	-	-	-	-
CD86	-	-	-	-	-
CD200R1	-	-	-	-	-
GDF11	yes	-	-	-	-
TNFRSF17	-	-	-	-	-
PROC	-	-	-	-	-
HDGFRP2	yes	-	-	-	-
ALCAM	-	-	-	-	-
CD109	-	-	-	-	-
CADM1	-	-	-	-	-
NOTCH1	-	-	-	-	-
IGF1R	-	-	-	-	-
MDH1	yes	-	-	yes	-
SEMA6A	-	-	-	-	-
PSPN	yes	-	-	-	-
TPM2	-	-	-	-	-
FAP	yes	-	-	-	-
EGFR	-	-	-	-	-
IL18BP	-	-	-	-	-
FASLG	-	-	-	-	-
