# Supplementary material for: Combined use of protein biomarkers and network analysis unveils deregulated regulatory circuits in Duchenne muscular dystrophy
Source: PLoS One. 2018 Mar 12;13(3):e0194225. doi: 10.1371/journal.pone.0194225 (PMC5846794; doi:10.1371/journal.pone.0194225)
Supplement: S5 Table — (PDF) [file pone.0194225.s005.pdf]

Table S5. Gene-set enrichment analysis using the protein signature in Hathout et al.

pathway source	ranking	pathway name	# genes in the signature	genes	p-value	BH adjusted p-value
biocarta	1	BIOCARTA SARS PATHWAY	3	CKM, GPT, LDHB	0.000255205	0.055379442
biocarta	2	BIOCARTA FIBRINOLYSIS PATHWAY	3	FGA, FGB, FGG	0.004594291	0.498480617
biocarta	3	BIOCARTA EXTRINSIC PATHWAY	3	FGA, FGB, FGG	0.010974765	0.60567573
biocarta	4	BIOCARTA AMI PATHWAY	3	FGA, FGB, FGG	0.01116453	0.60567573
biocarta	5	BIOCARTA INTRINSIC PATHWAY	3	FGA, FGB, FGG	0.017470808	0.758233056
biocarta	6	BIOCARTA KREB PATHWAY	1	MDH1	0.041255605	1
biocarta	7	BIOCARTA PROTEASOME PATHWAY	1	PSMA2	0.120701763	1
biocarta	8	BIOCARTA RHO PATHWAY	1	GSN	0.121125011	1
biocarta	9	BIOCARTA SET PATHWAY	1	SET	0.155284288	1
biocarta	10	BIOCARTA GLYCOLYSIS PATHWAY	1	GPI	0.189945879	1
KEGG	1	KEGG PYRUVATE METABOLISM	2	MDH1, LDHB	0.005136974	0.763515876
KEGG	2	KEGG ARGININE AND PROLINE METABOLISM	2	CKM, ACY1	0.016561432	0.763515876
KEGG	3	KEGG OLFACTORY TRANSDUCTION	2	CAMK2A, PRKACA	0.021109432	0.763515876
KEGG	4	KEGG NOTCH SIGNALING PATHWAY	2	JAG1, NOTCH3	0.032410539	0.763515876
KEGG	5	KEGG GLYOXYLATE AND DICARBOXYLATE METABOLISM	1	MDH1	0.041553749	0.763515876
KEGG	6	KEGG CITRATE CYCLE TCA CYCLE	1	MDH1	0.042163153	0.763515876
KEGG	7	KEGG PROPANOATE METABOLISM	1	LDHB	0.042201835	0.763515876
KEGG	8	KEGG STARCH AND SUCROSE METABOLISM	1	GPI	0.042950514	0.763515876
KEGG	9	KEGG TASTE TRANSDUCTION	1	PRKACA	0.042950514	0.763515876
KEGG	10	KEGG GLYCOLYSIS GLUCONEOGENESIS	2	GPI, LDHB	0.043074044	0.763515876
Reactome	1	REACTOME GLUCONEOGENESIS	3	GPI, MDH1, PRKACA	0.003345861	0.863673332
Reactome	2	REACTOME GRB2 SOS PROVIDES LINKAGE TO MAPK SIGNALING FOR INTERGRINS	3	FGA, FGB, FGG	0.004629262	0.863673332
Reactome	3	REACTOME GLUCOSE	3	GPI, MDH1,	0.005324149	0.863673332

		METABOLISM		PRKACA		
Reactome	4	REACTOME COMMON PATHWAY	3	FGA, FGB, FGG	0.006391066	0.863673332
Reactome	5	REACTOME P130CAS LINKAGE TO MAPK SIGNALING FOR INTEGRINS	3	FGA, FGB, FGG	0.006407072	0.863673332
Reactome	6	REACTOME STRIATED MUSCLE CONTRACTION	2	TNNI2, TNNI3	0.009750179	1
Reactome	7	REACTOME SIGNALING BY NOTCH3	2	JAG1, NOTCH3	0.015385376	1
Reactome	8	REACTOME INTEGRIN ALPHAIIB BETA3 SIGNALING	3	FGA, FGB, FGG	0.017639993	1
Reactome	9	REACTOME MUSCLE CONTRACTION	2	TNNI2, TNNI3	0.023872475	1
Reactome	10	REACTOME RECEPTOR LIGAND BINDING INITIATES THE SECOND PROTEOLYTIC CLEAVAGE OF NOTCH RECEPTOR	2	JAG1, NOTCH3	0.030542321	1
MSigDB hallmark collection	1	HALLMARK MYOGENESIS	6	TNNI2, FABP3, CKM, MAPK12, MB, GSN	0.004986277	0.133309521
MSigDB hallmark collection	2	HALLMARK OXIDATIVE PHOSPHORYLATION	3	GPI, MDH1, LDHB	0.005332381	0.133309521
MSigDB hallmark collection	3	HALLMARK NOTCH SIGNALING	2	JAG1, NOTCH3	0.031589493	0.526491545
MSigDB hallmark collection	4	HALLMARK WNT BETA CATENIN SIGNALING	1	JAG1	0.22917668	1
MSigDB hallmark collection	5	HALLMARK UV RESPONSE UP	2	PRKACA, RET	0.291353281	1
MSigDB hallmark collection	6	HALLMARK SPERMATOGENESIS	1	TNNI3	0.347750343	1
MSigDB hallmark collection	7	HALLMARK HEDGEHOG SIGNALING	1	UNC5C	0.349867886	1
MSigDB hallmark collection	8	HALLMARK INTERFERON ALPHA RESPONSE	1	CXCL10	0.366603542	1
MSigDB hallmark collection	9	HALLMARK KRAS SIGNALING DN	2	TNNI3, CKM	0.380315592	1
MSigDB hallmark collection	10	HALLMARK CHOLESTEROL HOMEOSTASIS	1	JAG1	0.387907404	1
