# Supplementary material for: Combined use of protein biomarkers and network analysis unveils deregulated regulatory circuits in Duchenne muscular dystrophy
Source: PLoS One. 2018 Mar 12;13(3):e0194225. doi: 10.1371/journal.pone.0194225 (PMC5846794; doi:10.1371/journal.pone.0194225)
Supplement: S6 Table — (PDF) [file pone.0194225.s006.pdf]

Table S6. NASFinder results obtained using the protein signature in Hathout et al.

pathway	regulator	# genes in the sub-network	# pathway overlapping genes	p-value
KEGG COMPLEMENT AND COAGULATION CASCADES	RXRA	61	4	0.00438532
KEGG GNRH SIGNALING	IRF3	62	7	5.30645E-05
KEGG MAPK SIGNALING	MEF2C	83	12	0.000163905
KEGG NOTCH SIGNALING	RBPJ	39	12	2.62209E-17
REACTOME IMMUNE SYSTEM	IRF3	62	25	6.82673E-07
REACTOME HEMOSTASIS	TP53	70	12	0.004442398
REACTOME METABOLISM OF CARBOHYDRATES	SMAD3	90	4	0.468561958
HALLMARK MYOGENESIS	MEF2C	83	9	0.001143349
HALLMARK COAGULATION	IRF3	62	3	0.153354909
HALLMARK UV RESPONSE UP	EIF2S3	46	3	0.106985225
BIOCARTA FIBRINOLYSIS	HIF1A	78	4	1.38E-05
BIOCARTA SARS	SMAD1	70	3	0.000198
