# Supplementary material for: Combined use of protein biomarkers and network analysis unveils deregulated regulatory circuits in Duchenne muscular dystrophy
Source: PLoS One. 2018 Mar 12;13(3):e0194225. doi: 10.1371/journal.pone.0194225 (PMC5846794; doi:10.1371/journal.pone.0194225)
Supplement: S7 Table — The top 20 pathways for each database are shown. (PDF) [file pone.0194225.s007.pdf]

Table S7. Pathway enrichment analysis of significantly enriched genes obtained from the transcriptomic dataset GDS3027. The top 20 pathways for each database are shown.

**Database**	**Gene Set Name**	**# Genes in Gene Set (K)**	**Description**	**# Genes in Overlap (k)**	**k/K**	**p-value**	**FDR q-value**
Reactome	REACTOME IMMUNE SYSTEM	933	Genes involved in Immune System	190	0.2036	2.24E-78	1.51E-75
Reactome	REACTOME ADAPTIVE IMMUNE SYSTEM	539	Genes involved in Adaptive Immune System	125	0.2319	3.26E-58	1.10E-55
Reactome	REACTOME HEMOSTASIS	466	Genes involved in Hemostasis	101	0.2167	2.49E-44	5.60E-42
Reactome	REACTOME CYTOKINE SIGNALING IN IMMUNE SYSTEM	270	Genes involved in Cytokine Signaling in Immune system	71	0.263	2.54E-37	3.98E-35
Reactome	REACTOME PLATELET ACTIVATION SIGNALING AND AGGREGATION	208	Genes involved in Platelet activation, signaling and aggregation	63	0.3029	2.95E-37	3.98E-35
Reactome	REACTOME DEVELOPMENTAL BIOLOGY	396	Genes involved in Developmental Biology	82	0.2071	1.14E-34	1.28E-32
Reactome	REACTOME AXON GUIDANCE	251	Genes involved in Axon guidance	61	0.243	3.58E-30	3.45E-28
Reactome	REACTOME CLASS I MHC MEDIATED ANTIGEN PROCESSING PRESENTATION	251	Genes involved in Class I MHC mediated antigen processing & presentation	59	0.2351	2.15E-28	1.81E-26
Reactome	REACTOME SIGNALING BY PDGF	122	Genes involved in Signaling by PDGF	42	0.3443	7.96E-28	5.96E-26
Reactome	REACTOME METABOLISM OF PROTEINS	518	Genes involved in Metabolism of proteins	82	0.1583	3.34E-26	2.25E-24
Reactome	REACTOME METABOLISM OF LIPIDS AND LIPOPROTEINS	478	Genes involved in Metabolism of lipids and lipoproteins	78	0.1632	7.44E-26	4.56E-24
Reactome	REACTOME INTEGRIN CELL SURFACE INTERACTIONS	79	Genes involved in Integrin cell surface interactions	32	0.4051	4.09E-24	2.30E-22

Reactome	REACTOME INTERFERON SIGNALING	159	Genes involved in Interferon Signaling	43	0.2704	1.12E-23	5.79E-22
Reactome	REACTOME RESPONSE TO ELEVATED PLATELET CYTOSOLIC CA2	89	Genes involved in Response to elevated platelet cytosolic Ca2+	33	0.3708	2.32E-23	1.12E-21
Reactome	REACTOME METABOLISM OF CARBOHYDRATES	247	Genes involved in Metabolism of carbohydrates	52	0.2105	8.00E-23	3.59E-21
Reactome	REACTOME ANTIGEN PROCESSING CROSS PRESENTATION	76	Genes involved in Antigen processing-Cross presentation	28	0.3684	5.98E-20	2.52E-18
Reactome	REACTOME SIGNALLING BY NGF	217	Genes involved in Signalling by NGF	45	0.2074	1.14E-19	4.51E-18
Reactome	REACTOME FATTY ACID TRIACYLGLYCEROL AND KETONE BODY METABOLISM	168	Genes involved in Fatty acid, triacylglycerol, and ketone body metabolism	39	0.2321	4.75E-19	1.78E-17
Reactome	REACTOME GLUCOSE METABOLISM	69	Genes involved in Glucose metabolism	26	0.3768	6.47E-19	2.30E-17
Reactome	REACTOME CELL SURFACE INTERACTIONS AT THE VASCULAR WALL	91	Genes involved in Cell surface interactions at the vascular wall	29	0.3187	1.34E-18	4.51E-17
KEGG	KEGG FOCAL ADHESION	201	Focal adhesion	68	0.3383	1.23E-43	2.29E-41
KEGG	KEGG REGULATION OF ACTIN CYTOSKELETON	216	Regulation of actin cytoskeleton	56	0.2593	2.27E-29	2.11E-27
KEGG	KEGG VIRAL MYOCARDITIS	73	Viral myocarditis	34	0.4658	4.99E-28	3.09E-26
KEGG	KEGG PATHWAYS IN CANCER	328	Pathways in cancer	66	0.2012	2.15E-27	1.00E-25
KEGG	KEGG ANTIGEN PROCESSING AND PRESENTATION	89	Antigen processing and presentation	36	0.4045	6.33E-27	2.35E-25
KEGG	KEGG CELL ADHESION MOLECULES CAMS	134	Cell adhesion molecules (CAMs)	41	0.306	6.33E-25	1.96E-23
KEGG	KEGG ECM RECEPTOR INTERACTION	84	ECM-receptor interaction	33	0.3929	2.60E-24	6.91E-23

KEGG	KEGG LEISHMANIA INFECTION	72	Leishmania infection	30	0.4167	3.94E-23	9.17E-22
KEGG	KEGG LEUKOCYTE TRANSENDOTHELIAL MIGRATION	118	Leukocyte transendothelial migration	37	0.3136	4.72E-23	9.75E-22
KEGG	KEGG LYSOSOME	121	Lysosome	36	0.2975	1.34E-21	2.49E-20
KEGG	KEGG MAPK SIGNALING PATHWAY	267	MAPK signaling pathway	51	0.191	1.95E-20	3.30E-19
KEGG	KEGG CHEMOKINE SIGNALING PATHWAY	190	Chemokine signaling pathway	43	0.2263	2.13E-20	3.30E-19
KEGG	KEGG INSULIN SIGNALING PATHWAY	137	Insulin signaling pathway	36	0.2628	1.32E-19	1.89E-18
KEGG	KEGG NEUROTROPHIN SIGNALING PATHWAY	126	Neurotrophin signaling pathway	34	0.2698	5.41E-19	7.19E-18
KEGG	KEGG ALZHEIMERS DISEASE	169	Alzheimer's disease	39	0.2308	5.93E-19	7.35E-18
KEGG	KEGG SMALL CELL LUNG CANCER	84	Small cell lung cancer	27	0.3214	1.59E-17	1.85E-16
KEGG	KEGG ALLOGRAFT REJECTION	38	Allograft rejection	19	0.5	5.17E-17	5.65E-16
KEGG	KEGG CALCIUM SIGNALING PATHWAY	178	Calcium signaling pathway	37	0.2079	1.81E-16	1.87E-15
KEGG	KEGG DILATED CARDIOMYOPATHY	92	Dilated cardiomyopathy	27	0.2935	2.15E-16	2.10E-15
KEGG	KEGG GLIOMA	65	Glioma	23	0.3538	3.44E-16	3.20E-15
Hallmark	HALLMARK EPITHELIAL MESENCHYMAL TRANSITION	200	Genes defining epithelial-mesenchymal transition, as in wound healing, fibrosis and metastasis.	82	0.41	3.34E-60	1.67E-58
Hallmark	HALLMARK MYOGENESIS	200	Genes involved in development of skeletal muscle (myogenesis).	73	0.365	1.92E-49	4.80E-48
Hallmark	HALLMARK INTERFERON GAMMA RESPONSE	200	Genes up-regulated in response to IFNG [GeneID=3458].	61	0.305	2.60E-36	4.34E-35

Hallmark	HALLMARK OXIDATIVE PHOSPHORYLATION	200	Genes encoding proteins involved in oxidative phosphorylation.	58	0.29	2.95E-33	3.69E-32
Hallmark	HALLMARK APICAL JUNCTION	200	Genes encoding components of apical junction complex.	56	0.28	2.84E-31	2.84E-30
Hallmark	HALLMARK APOPTOSIS	161	Genes mediating programmed cell death (apoptosis) by activation of caspases.	50	0.3106	1.99E-30	1.66E-29
Hallmark	HALLMARK COMPLEMENT	200	Genes encoding components of the complement system, which is part of the innate immune system.	55	0.275	2.69E-30	1.92E-29
Hallmark	HALLMARK ADIPOGENESIS	200	Genes up-regulated during adipocyte differentiation (adipogenesis).	54	0.27	2.47E-29	1.37E-28
Hallmark	HALLMARK ALLOGRAFT REJECTION	200	Genes up-regulated during transplant rejection.	54	0.27	2.47E-29	1.37E-28
Hallmark	HALLMARK COAGULATION	138	Genes encoding components of blood coagulation system; also up-regulated in platelets.	45	0.3261	1.50E-28	7.49E-28
Hallmark	HALLMARK FATTY ACID METABOLISM	158	Genes encoding proteins involved in metabolism of fatty acids.	47	0.2975	9.34E-28	4.18E-27
Hallmark	HALLMARK INTERFERON ALPHA RESPONSE	97	Genes up-regulated in response to alpha interferon proteins.	38	0.3918	1.00E-27	4.18E-27
Hallmark	HALLMARK GLYCOLYSIS	200	Genes encoding proteins involved in glycolysis and gluconeogenesis.	48	0.24	8.73E-24	3.36E-23
Hallmark	HALLMARK HYPOXIA	200	Genes up-regulated in response to low oxygen levels (hypoxia).	47	0.235	6.68E-23	2.23E-22
Hallmark	HALLMARK IL2 STAT5 SIGNALING	200	Genes up-regulated by STAT5 in response to IL2 stimulation.	47	0.235	6.68E-23	2.23E-22
Hallmark	HALLMARK KRAS	200	Genes up-regulated	46	0.23	4.97E-	1.55E-21

	SIGNALING UP		by KRAS activation.			22	
Hallmark	HALLMARK INFLAMMATORY RESPONSE	200	Genes defining inflammatory response.	44	0.22	2.52E-20	7.42E-20
Hallmark	HALLMARK UV RESPONSE DN	144	Genes down-regulated in response to ultraviolet (UV) radiation.	37	0.2569	9.50E-20	2.64E-19
Hallmark	HALLMARK P53 PATHWAY	200	Genes involved in p53 pathways and networks.	43	0.215	1.72E-19	4.53E-19
Hallmark	HALLMARK MTORC1 SIGNALING	200	Genes up-regulated through activation of mTORC1 complex.	42	0.21	1.14E-18	2.71E-18
Biocarta	BIOCARTA BIOPEPTIDES PATHWAY	45	Bioactive Peptide Induced Signaling Pathway	17	0.3778	7.14E-13	1.55E-10
Biocarta	BIOCARTA FCER1 PATHWAY	41	Fc Epsilon Receptor I Signaling in Mast Cells	15	0.3659	2.78E-11	3.02E-09
Biocarta	BIOCARTA HIVNEF PATHWAY	58	HIV-I Nef: negative effector of Fas and TNF	17	0.2931	7.76E-11	5.39E-09
Biocarta	BIOCARTA PGC1A PATHWAY	26	Regulation of PGC-1a	12	0.4615	1.04E-10	5.39E-09
Biocarta	BIOCARTA HDAC PATHWAY	32	Control of skeletal myogenesis by HDAC and calcium/calmodulin-dependent kinase (CaMK)	13	0.4062	1.24E-10	5.39E-09
Biocarta	BIOCARTA NFAT PATHWAY	56	NFAT and Hypertrophy of the heart (Transcription in the broken heart)	16	0.2857	4.26E-10	1.54E-08
Biocarta	BIOCARTA EIF4 PATHWAY	24	Regulation of eIF4e and p70 S6 Kinase	11	0.4583	6.99E-10	2.17E-08
Biocarta	BIOCARTA BCR PATHWAY	37	BCR Signaling Pathway	13	0.3514	1.06E-09	2.86E-08
Biocarta	BIOCARTA MAPK PATHWAY	87	MAPKinase Signaling Pathway	19	0.2184	1.54E-09	3.71E-08
Biocarta	BIOCARTA MTOR PATHWAY	23	mTOR Signaling Pathway	10	0.4348	8.00E-09	1.74E-07
Biocarta	BIOCARTA CLASSIC PATHWAY	14	Classical Complement Pathway	8	0.5714	1.69E-08	3.11E-07

Biocarta	BIOCARTA COMP PATHWAY	19	Complement Pathway	9	0.4737	1.80E-08	3.11E-07
Biocarta	BIOCARTA IL2RB PATHWAY	38	IL-2 Receptor Beta Chain in T cell Activation	12	0.3158	1.86E-08	3.11E-07
Biocarta	BIOCARTA IGF1MTOR PATHWAY	20	Skeletal muscle hypertrophy is regulated via AKT/mTOR pathway	9	0.45	3.16E-08	4.90E-07
Biocarta	BIOCARTA CDC42RAC PATHWAY	16	Role of PI3K subunit p85 in regulation of Actin Organization and Cell Migration	8	0.5	6.74E-08	9.75E-07
Biocarta	BIOCARTA AT1R PATHWAY	36	Angiotensin II mediated activation of JNK Pathway via Pyk2 dependent signaling	11	0.3056	1.07E-07	1.46E-06
Biocarta	BIOCARTA IGF1R PATHWAY	23	Multiple antiapoptotic pathways from IGF-1R signaling lead to BAD phosphorylation	9	0.3913	1.38E-07	1.76E-06
Biocarta	BIOCARTA PTEN PATHWAY	18	PTEN dependent cell cycle arrest and apoptosis	8	0.4444	2.13E-07	2.57E-06
Biocarta	BIOCARTA FMLP PATHWAY	39	fMLP induced chemokine gene expression in HMC-1 cells	11	0.2821	2.68E-07	3.06E-06
Biocarta	BIOCARTA RHO PATHWAY	32	Rho cell motility signaling pathway	10	0.3125	3.23E-07	3.51E-06
