# Supplementary material for: Combined use of protein biomarkers and network analysis unveils deregulated regulatory circuits in Duchenne muscular dystrophy
Source: PLoS One. 2018 Mar 12;13(3):e0194225. doi: 10.1371/journal.pone.0194225 (PMC5846794; doi:10.1371/journal.pone.0194225)
Supplement: S1 Fig — (PDF) [file pone.0194225.s008.pdf]

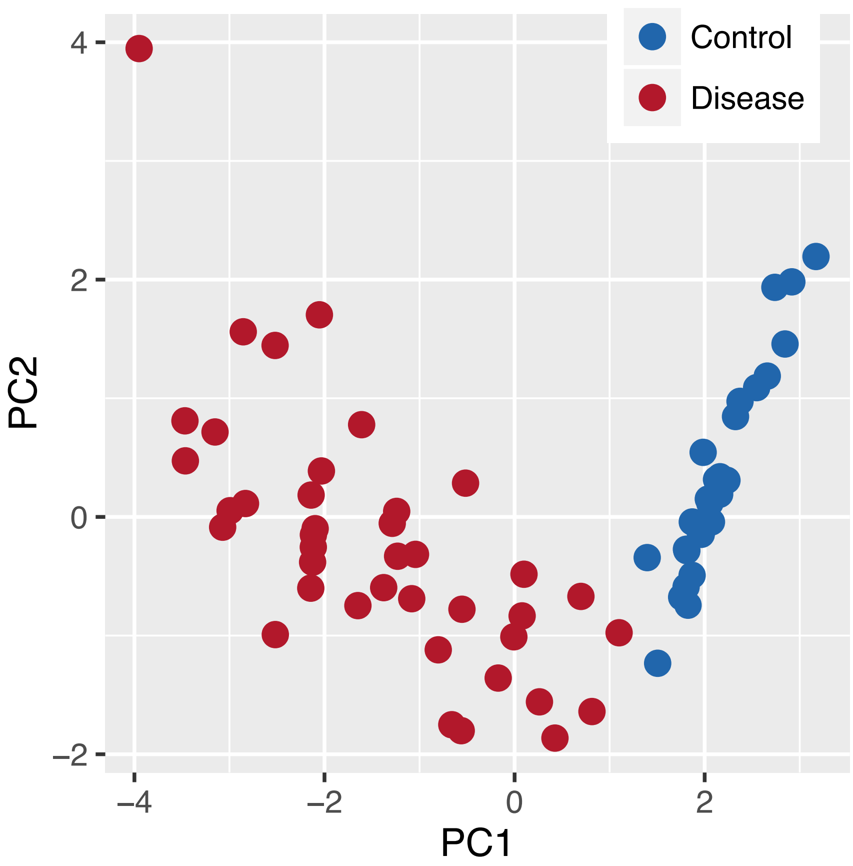

Figure S1. Principal Component Analysis of a subset of the DMD dataset that includes only the 6 proteins in the short biomarker panel.
