# Supplementary material for: Combined use of protein biomarkers and network analysis unveils deregulated regulatory circuits in Duchenne muscular dystrophy
Source: PLoS One. 2018 Mar 12;13(3):e0194225. doi: 10.1371/journal.pone.0194225 (PMC5846794; doi:10.1371/journal.pone.0194225)
Supplement: S2 Fig — The plots show all the proteins in the long biomarker panel showing a significant correlation with age. (PDF) [file pone.0194225.s009.pdf]

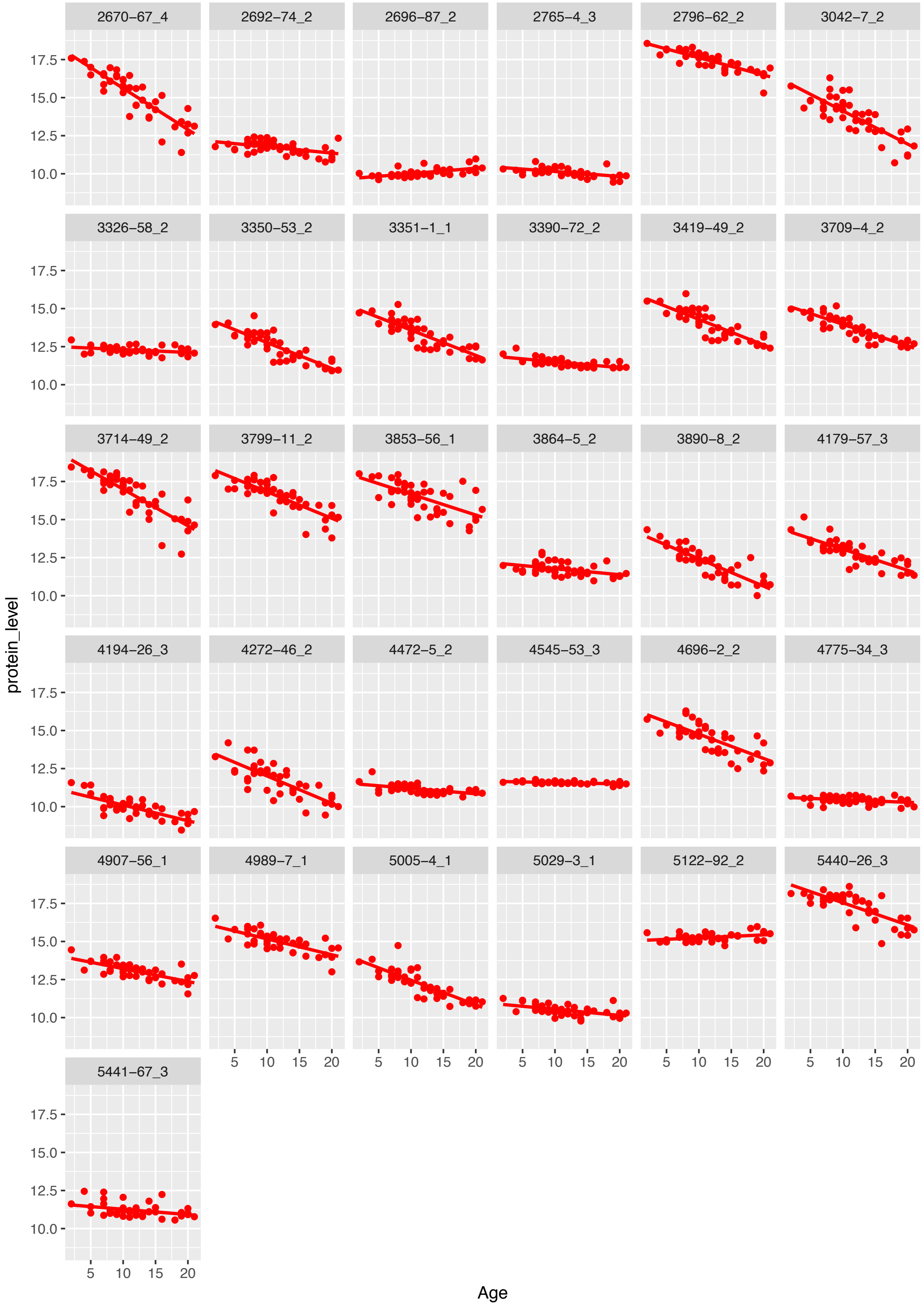

Figure S2. Age-related protein level variation in DMD subjects. The plots show all the proteins in the long biomarker panel showing a significant correlation with age.
