# Supplementary material for: Combined use of protein biomarkers and network analysis unveils deregulated regulatory circuits in Duchenne muscular dystrophy
Source: PLoS One. 2018 Mar 12;13(3):e0194225. doi: 10.1371/journal.pone.0194225 (PMC5846794; doi:10.1371/journal.pone.0194225)
Supplement: S4 Fig — Only the proteins in the long biomarker panel with a significant difference are shown. (PDF) [file pone.0194225.s011.pdf]

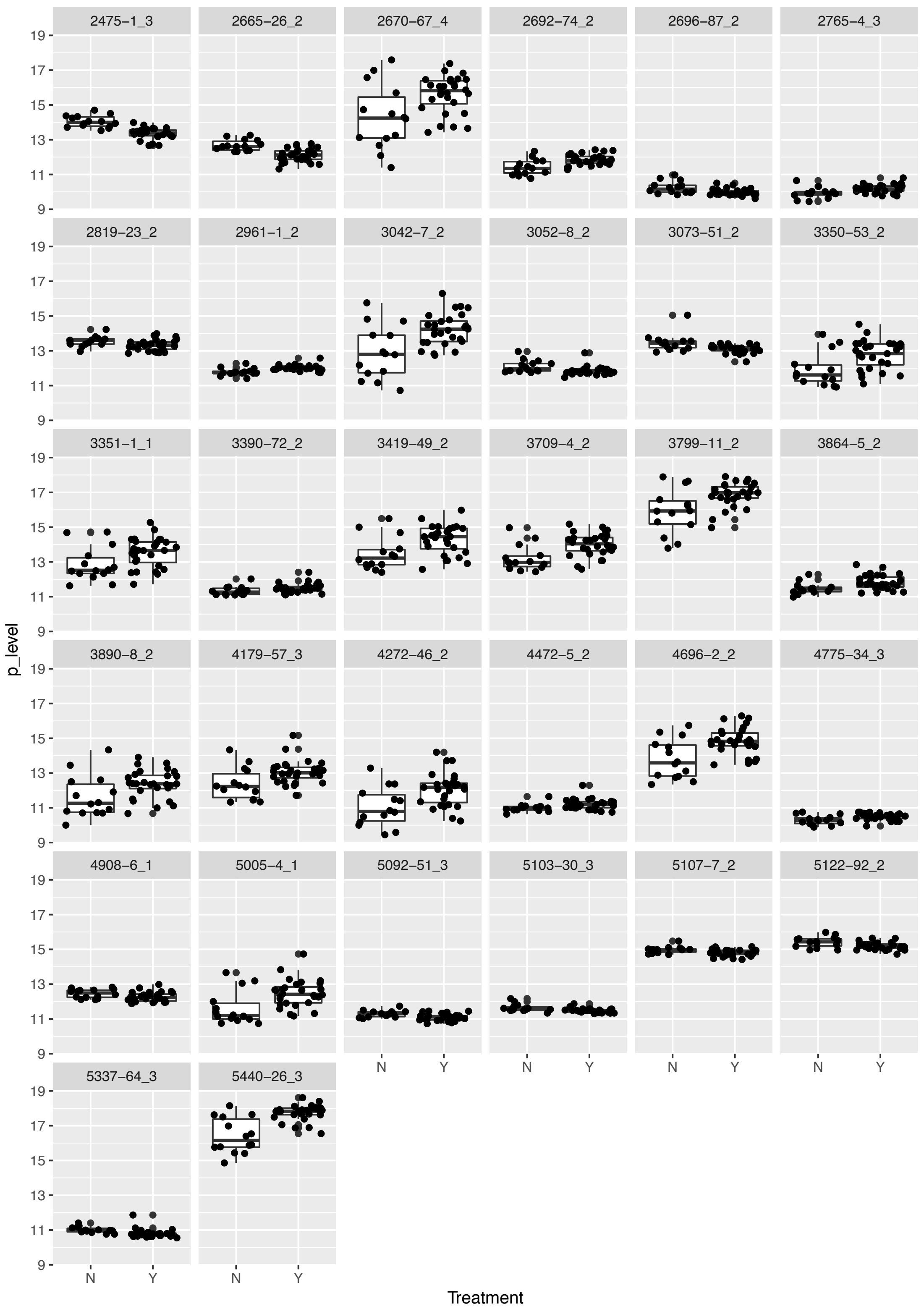

Figure S4. Charts showing the treatment effect in patients. Only the proteins in the long biomarker panel with a significant difference are shown.
