# Supplementary material for: Combined use of protein biomarkers and network analysis unveils deregulated regulatory circuits in Duchenne muscular dystrophy
Source: PLoS One. 2018 Mar 12;13(3):e0194225. doi: 10.1371/journal.pone.0194225 (PMC5846794; doi:10.1371/journal.pone.0194225)
Supplement: S5 Fig — The nodes of the network represent the samples, while colors indicate their status (controls, disease without treatment, diseases with treatment). The length of the edges is proportional to level of similarity between the sample signatures. In both cases we can observe that controls and disease samples cluster together forming two separate groups, while this does not happen if we consider the treatment (red nodes and orange ones are not clearly separated). (A) Short biomarker panel. (B) Long biomarker panel. (PDF) [file pone.0194225.s012.pdf]

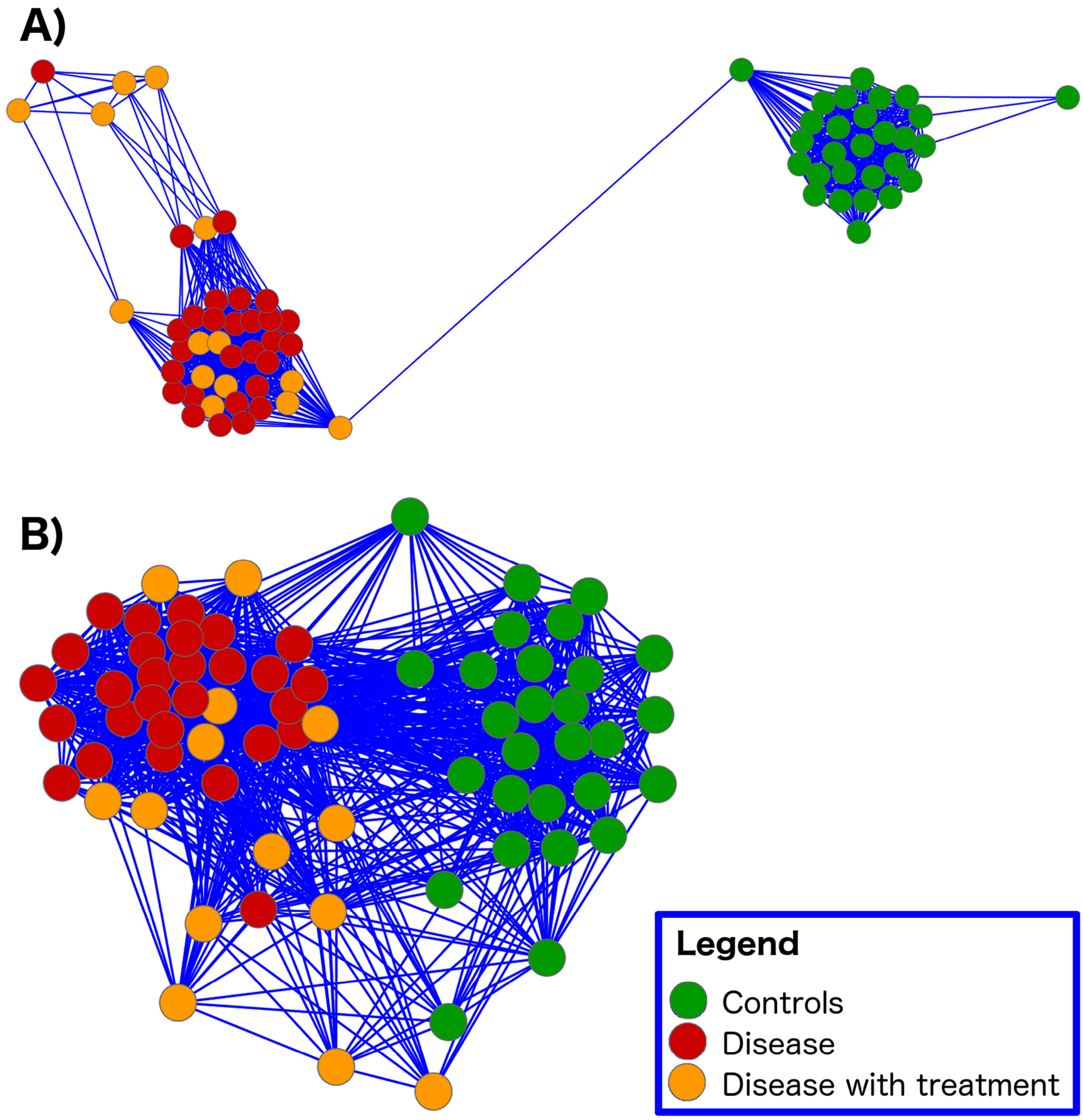

S5 Figure. Network plots showing the case control differentiation using the proteins in the biomarker panels. The nodes of the network represent the samples, while colors indicate their status (controls, disease without treatment, diseases with treatment). The length of the edges is proportional to level of similarity between the sample signatures. In both cases we can observe that controls and disease samples cluster together forming two separate groups, while this does not happen if we consider the treatment (red nodes and orange ones are not clearly separated). (A) Short biomarker panel. (B) Long biomarker panel.
